# Supplementary material for: Study protocol for ‘The Project About Loneliness and Social networks (PALS)’: a pragmatic, randomised trial comparing a facilitated social network intervention (Genie) with a wait-list control for lonely and socially isolated people
Source: BMJ Open. 2019 Aug 18;9(8):e028718. doi: 10.1136/bmjopen-2018-028718 (PMC6701612; doi:10.1136/bmjopen-2018-028718)
Supplement: Author's manuscript [file bmjopen-2018-028718.draft_revisions.pdf]

BMJ Open is committed to open peer review. As part of this commitment we make the peer review history of every article we publish publicly available.

When an article is published we post the peer reviewers' comments and the authors' responses online. We also post the versions of the paper that were used during peer review. These are the versions that the peer review comments apply to.

The versions of the paper that follow are the versions that were submitted during the peer review process. They are not the versions of record or the final published versions. They should not be cited or distributed as the published version of this manuscript.

BMJ Open is an open access journal and the full, final, typeset and author-corrected version of record of the manuscript is available on our site with no access controls, subscription charges or pay-per-view fees (http://bmjopen.bmj.com).

If you have any questions on BMJ Open's open peer review process please email info.bmjopen@bmj.com

BMJ Open

Study protocol for 'The Project About Loneliness and Social networks (PALS)': a pragmatic, randomised trial comparing a facilitated social network intervention (Genie) with a wait-list control for lonely and socially-isolated people

Journal:	*BMJ Open*
Manuscript ID	bmjopen-2018-028718
Article Type:	Protocol
Date Submitted by the Author:	20-Dec-2018
Complete List of Authors:	Band, Rebecca; University of Southampton, Psychology ; University of Southampton, Health Sciences<br>Ewings, Sean; University of Southampton, Health Sciences<br>Cheetham-Blake, Tara; University of Southampton, Faculty of Health Sciences<br>Ellis, Jaimie; University of Southampton, Health Sciences<br>Breheny, Katie; University of Bristol, Population Health Sciences<br>Vassilev, Ivaylo; University of Southampton, Health Sciences<br>Portillo , Mari Carmen; University of Southampton, Health Sciences<br>Yardley, Lucy; University of Southampton, Academic Unit of Psychology; University of Bristol, School of Psychological Science<br>Blickem, C; Liverpool John Moores University, Public Health<br>Kandiyali, Rebecca; University of Bristol School of Social and Community Medicine, Centre for Child and Adolescent Health<br>Culliford, David; University of Southampton, Faculty of Medicine<br>Rogers, Anne; University of Southampton, Faculty of Health Sciences
Keywords:	Protocol, Randomised Controlled Trial, Social networks, Social isolation, Loneliness

SCHOLARONE™
Manuscripts

Study protocol for 'The Project About Loneliness and Social networks (PALS)': a pragmatic, randomised trial comparing a facilitated social network intervention (Genie) with a wait-list control for lonely and socially-isolated people

Protocol version: 1

This corresponds with the study protocol version 4.

Rebecca Band[1,2], Sean Ewings[1], Tara Cheetham-Blake[1], Jaimie Ellis[1], Katie Breheny[3], Ivaylo Vassilev[1], Mari Carmen Portillo[1], Lucy Yardley[2, 4], Christian Blickem[5], Rebecca Kandiyali[3], David Culliford[1], Anne Rogers[1]

[1] Health Sciences, University of Southampton, Southampton, UK

[2] Academic unit of psychology, University of Southampton, Southampton, UK

[3] Population Health Sciences, Bristol Medical School, University of Bristol, UK

[4] School of Psychological Science, University of Bristol, UK

[5] Public Health Institute, Faculty of Health, Education and Community, Liverpool John Moores University, Liverpool, UK

Corresponding author: Rebecca Band, Highfield Campus, University of Southampton, Southampton. SO17 1BJ.

Keywords: Loneliness, Social isolation, Social networks, Randomised Controlled Trial, Protocol

Word count: 4234

For peer review only - http://bmjopen.bmj.com/site/about/guidelines.xhtml

Abstract:

Introduction:

Loneliness and social isolation have been identified as significant public health concerns, but improving relationships and increasing social participation may improve health outcomes and quality of life. The aim of the PALS study is to assess the effectiveness and cost-effectiveness of the Genie intervention within a community setting among individuals at-risk of loneliness and isolation and to understand implementation of Genie in the context of different organisations.

Methods and analysis:

The PALS trial will be a pragmatic, randomised controlled trial comparing participants receiving the Genie intervention to a wait-list control group. Eligible participants will be recruited from organisations working within a community setting: any adult identified as socially isolated or at-risk of loneliness and living in the community will be eligible. Genie will be delivered by trained facilitators recruited from community organisations. The primary outcome will be the difference in the SF-12 Mental Health composite scale score at 6-month follow-up between the intervention and control group using a mixed effects model (accounting for clustering within facilitators and organisation). Secondary outcomes will be loneliness; social isolation; wellbeing; physical health and engagement with new activities. The economic evaluation will use a cost-utility approach, and adopt a public sector perspective to include health-related resource use and costs incurred by other public services. Exploratory analysis will use a societal perspective, and explore broader measures of benefit (capability wellbeing). A qualitative process evaluation will explore organisational and environmental arrangements, as well as stakeholder and participant experiences of the study

For peer review only - http://bmjopen.bmj.com/site/about/guidelines.xhtml

to understand the factors likely to influence future sustainability, implementation and scalability of using a social network intervention within this context.

Ethics and dissemination:

This study has received NHS ethical approval (REC reference: 18/SC/0245). The findings from PALS will be disseminated widely through peer-reviewed publications, conferences and workshops in collaboration with our community partners.

Trial registration number: ISRCTN 19193075

299/300 words

Strengths and limitations

- This study will evaluate an existing social network intervention (Genie) in the context of loneliness and social isolation
- The PALS study consists of a pragmatic RCT implemented in conjunction with community-based stakeholders in a community setting in two areas of the UK
- The process evaluation and analysis has been designed to understand the factors influencing the implementation and scalability of social network interventions in this context

For peer review only

For peer review only - http://bmjopen.bmj.com/site/about/guidelines.xhtml

INTRODUCTION

Social isolation is considered to be an objective lack of social connections, contact or participation, while loneliness is a subjective psychological state where there is a discrepancy between desired and perceived levels of support or connectedness [1, 2]. The prevalence rates of loneliness and isolation vary [3], however it is estimated to affect about 30% of the adult population in the UK [4]. Specific at-risk groups, such as the elderly, minority communities, and those with long-term mental or physical health conditions are significantly more isolated than those in good health [3, 5, 6]. The Office of National Statistics (ONS) recently identified three profiles of individuals who are 'at-risk'; these suggest different factors may be important at different points across the life-course [7].

The problem: health implications of loneliness and social isolation

The impact of loneliness and isolation on well-being and the associated health risks have been identified as a significant public health concern [8, 9], exacerbated by the prevalence of long-term conditions and advancing age [10]. Both loneliness and social isolation are associated with poor physical and mental health outcomes [11-13], reduced quality of life [14, 15] as well as being linked to poorer physiological outcomes such as raised blood pressure and increased health-risk behaviours (e.g. sedentary behaviour) [16]. Their impact on mortality is estimated to exceed that of traditional risk factors such as obesity and cigarette smoking, with a 50% higher risk compared with socially-integrated participants [17-19]. There are also significant costs associated with raised demand and use of health services, and loneliness is associated with increased GP appointments, emergency hospital admittance and premature social care use [20-22].

Social relationships and preventing or reducing loneliness and social isolation

For peer review only - http://bmjopen.bmj.com/site/about/guidelines.xhtml

Although the determinants of loneliness and isolation are varied, social and emotional support from others is likely to be protective [23], with emerging evidence suggesting that improving the quality of interpersonal relationships and participation in social activities may be key to tackling the impact of loneliness [9]. Evidence has indicated that increasing social interactions and the number of people who can be relied on is associated with reduced levels of distress [24], whilst connecting with community resources can help protect against loneliness for those who are most at risk [9, 25]. Furthermore, there is evidence that social network interventions can significantly improve health outcomes, quality of life and increase the take-up of new activities [26, 27]. A diverse and supportive network has been shown to reduce health service costs [28]. A recent NICE quality standard recommends the navigation of older vulnerable people to community activities as a means of preventing loneliness in this group [25].

Rationale and risk-benefits for the current trial

In line with this evidence, there is a logical argument for introducing an effective social network intervention outside of formal healthcare settings to connect people who are at risk of loneliness to others within their communities [25]. Creative engagement with non-traditional informal providers of wellness management (such as through accessing locally available community groups) offers an alternative opportunity to address health and social needs. A series of nestled qualitative process studies will examine the context, practices and processes relating to implementing the intervention within the community context, and an economic evaluation to assess whether this is cost-effective.

Study aims and research questions

For peer review only - http://bmjopen.bmj.com/site/about/guidelines.xhtml

The aim of the PALS study is to assess the feasibility, acceptability, effectiveness and cost-effectiveness of a facilitated social network intervention (Genie) compared to a wait-list control within a community setting among at-risk populations, and to understand the implementation in the context of different organisations who work in this environment.

Primary objectives

- To determine the effect of Genie compared to usual care on mental health at three and six months.

Secondary objectives

- To determine the effect of Genie compared to usual care on loneliness, social isolation, physical health, and engagement with new activities at three and six months.
- To establish whether the use of Genie within a community setting is cost-effective.

Process analysis objectives

- To assess the acceptability and feasibility of running the study based on recruitment and retention during an internal pilot phase.
- To explore the experiences of using Genie, how the intervention impacts on loneliness and isolation, and the mechanisms by which participants enact change.
- To explore contextual environmental and organisational factors that inhibit or promote the integration, sustainability and scalability of Genie for addressing loneliness in local and organisational settings.

METHODS AND ANALYSIS

Study design and setting

We will conduct a pragmatic, randomised controlled trial comparing participants receiving the facilitated social network Genie intervention to a wait-list control group; randomisation

For peer review only - http://bmjopen.bmj.com/site/about/guidelines.xhtml

will be at individual and/or cluster (facilitator) level (see Randomisation section). We will work closely with community partners two localities (centred around Southampton and Liverpool) in identifying participants and delivering the intervention, as well as informing our understanding of the challenges and environmental factors associated with implementation. Partners may include any group or organisation that has the potential to identify or access at-risk individuals.

Study participants

Identification

We will use a multi-stranded recruitment strategy to reflect the diversity of individuals who are living with loneliness or in isolation. This will be facilitated by collaborating community organisations to ensure that we are able to identify and access those most at-risk. Potential participants will be identified in the manner that best operates within existing working practices for each organisation (which will be different for each organisation/ collaborator). This is necessary to explore the integration and scalability of Genie in local and organisational settings. Potential participants will be invited by the organisation; this may be by letter or during routine visits, appointments, or in line with the usual working practices of the partner organisation. All eligible participants will be given a research pack including an invitation letter, participant information sheet, and freepost reply slip to return should they wish to take part in the trial.

Eligibility criteria

We will recruit any adult (aged 18 or over) who is identified as being isolated or at risk of loneliness. We define a socially isolated person as one for whom there is an "absence of social contacts or community involvement, or lack of access to services" in line with the definition used by Hampshire County Council [29].

For peer review only - http://bmjopen.bmj.com/site/about/guidelines.xhtml

Exclusion Criteria

Exclusion criteria will include participants who are currently hospitalised (i.e. not self-managing within a community setting), those in the end stages of life or any condition which impacts upon ability to take part, those lacking sufficient capacity and those having previously used the Genie intervention.

Randomisation

The randomisation process is partly determined by the structure of each organisation and is designed to ensure that a) the risk of contamination across study arms is minimised, and b) allocation concealment is maintained. Facilitators will be randomised (1:1) to either the intervention or control arm where possible, and participants also randomly allocated (1:1) to the corresponding arm via an independent process within each organisation. In this case, facilitator randomisation will be stratified by organisation with blocks of two (i.e., one facilitator will be randomised to the intervention arm and one to the control arm) and conducted by the trial statistician (SE). Where there are practical constraints on facilitators who work within a specific locality (i.e. geographical, services or otherwise), the facilitator will be randomised (1:1) to either the intervention or control arm but participants within the locality will not be randomised. In instances where facilitators do not have an ongoing relationship with potential participants, and none of the above constraints apply, only participants will be randomised (1:1). Blocking will occur in all cases. Randomisation sequences will be computer-generated. The sequences will be stored in sealed, opaque, numbered envelopes. For facilitators within an organisation, assignment to intervention or control will happen simultaneously once they have agreed to take part in the study.

Participant flow through the study

For peer review only - http://bmjopen.bmj.com/site/about/guidelines.xhtml

Written informed consent will be sought from all participants and baseline data collected with a research team member (online or on paper, dependent on the participant preferences). Allocation will occur once the baseline assessment has been completed. Participants who are allocated to the intervention condition will be given access to the Genie intervention within two weeks of the baseline appointment; this process will be guided by the facilitator at a location to suit them (i.e. at home or in the community). At 3 and 6 months after enrolment into the study, participants will be invited to complete follow-up assessments. All follow-up assessments will be recorded no earlier than two weeks before the follow-up date and no later than six weeks after the follow-up date. Each participant will be sent a £10 high street gift voucher with the 6-month follow-up questionnaire. Individuals allocated to the control group will be offered access to Genie with the facilitator after the have completed their 6-month follow-up assessment.

Sample size consideration

The sample size calculation is based on the primary analysis of the comparison of intervention and control arms on SF-12 Mental Health composite scale score at six months [30], and accounts for possible intra-cluster correlation (ICC) within facilitators. Previous studies (albeit in different populations) have suggested that differences of 3 and 4.7 points on the SF-12 would be clinically meaningful [31, 32]. We have based the current sample size on being able to detect a difference of 4 points. Based on a previous study in socially-isolated older people [33], we estimate the standard deviation of the outcome to be 10.4 (using a pooled estimate of baseline scores). Choosing 80% power and a type I error rate of 5%, an individually-randomised study would require 216 people (108 per arm). Regarding clustering, previous studies have generally shown low ICCs for mental health scores from SF-12 and SF-36 (0.032 and below, albeit for different populations and clustering within GP practices) [33, 34]; we use an ICC of 0.05 here. Based on discussions with participating organisations, it was

For peer review only - http://bmjopen.bmj.com/site/about/guidelines.xhtml

agreed that 12 participants per facilitator was suitable; this results in a design effect of 1.55 and an adjusted sample size of 335 people. Assuming 15% drop-out [35], we require 394 participants in total (197 per arm). This requires 33 facilitators; we will increase this to 36 facilitators to account for potential drop-out of facilitators.

The facilitated Social network Intervention

The Genie (Generating Engagement in Network Involvement) intervention is an online, facilitated, social networking tool designed to develop opportunities for social involvement (https://pals.genie-net.org/eng/), which by design, can be applied to varied user groups [27]. It is based on evidence of social network properties and types, mechanisms and work relating to managing health and wellness [36-39]. The intervention process is introduced initially via a guided discussion with a trained facilitator, takes 30 to 40 minutes to deliver and has three stages: social network mapping, tailoring of preferences, linking users to valued resources and activities. Previous testing of the principles has shown that it is both appropriate and acceptable to implement for individuals with a long- term condition [26-28].

Facilitators

Guided facilitation is an important element of the guided process to using the tool. Facilitators do not need a specific in-depth theoretical knowledge: instead, the local knowledge of facilitators is important and adds to the value of the intervention. However, the interpersonal skills of the facilitator are vital for the success of engagement through promoting a collaborative solution, and engaging participant focus, motivation and reflection on social network composition and promoting new community engagement [27]. Facilitators receive a minimum of a half-day training from the research team, which may be refreshed over the course of the study. This will include a background to, demonstration of, and practical pair-working exercises using video guides around the facilitation process. Research

For peer review only - http://bmjopen.bmj.com/site/about/guidelines.xhtml

methods training and discussion around loneliness and isolation are also addressed. The research team will provide ongoing support to monitor fidelity to the intervention deployment and address issues arising regarding complex cases (or facilitator difficulties and distress).

Social network mapping

Facilitators guide participants to create a visual map of their current support network, using a concentric circles method [27]. The concentric circles process provides insight into the user's current situation regarding social support; who they view as important in their daily lives (this may include family members, friends, acquaintances, healthcare professionals, local groups and pets); and then to reflect on renegotiating existing roles and responsibilities, and further map people and groups who could provide extended support [26-28]. This process, when guided by the facilitator, helps the participant to realign thinking about their relationships (and conceptualise themselves within a network of support), explore family dynamics and recognise 'weak ties' (i.e. social acquaintances) that already exist in their network [27]. It also offers the opportunity to begin discussions about how support may be extended within the network.

Linking individuals with preferences and valued local and online activities and resources

The next step involves facilitating access to local resources based on personal preferences, and acceptability to encourage engagement with personal choices, through a set of 13 questions [40]. The questions generate a set of preferred local and online resources (linked to a pre-created database of categorised local organisations and resources). The facilitated discussion of preferences is linked to available and accepted potential support from people in a person's network. Personalised results are presented in a user-friendly way aided by Google maps with clear details about access. Previous work has highlighted that this is often new and previously un-thought about information for participants [27]. The network maps, description

For peer review only - http://bmjopen.bmj.com/site/about/guidelines.xhtml

of individual networks, preferences, and the local and online resources identified as relevant by individuals can be printed to keep or re-accessed online later via a personalised Genie page [40, 41]. Two weeks after the intervention all Genie users receive a phone call from the facilitator and alternative or additional engagement activities are discussed. The follow up call takes up to 10-15 minutes.

Wait-list control group

All participants allocated to the control group will be offered the opportunity to use the Genie intervention with a facilitator once the 6-month follow-up has been completed to avoid increasing inequalities as a result of the study, particularly for participants living in marginalized and deprived domestic situations.

Outcomes

The primary outcome of the trial will be difference in the SF-12 Mental Health composite scale score at 6-month follow-up between the intervention and control group using a mixed effects model (accounting for clustering within facilitators and organisation).

Secondary outcomes will include:

- Difference in the SF-12 Mental Health composite scale score between the intervention and control group at 3-month follow-up
- Difference in SF-12 Physical Health composite score between intervention and control groups at 6-month follow-up
- Difference in loneliness between intervention and control groups at 3 and 6-month follow-up measured using the De Jong Loneliness scale [42] and the Campaign against loneliness measure [43]

For peer review only

- Difference in social isolation between intervention and control groups at 3 and 6-month follow-up measured using the Duke Social Support index [44]
- Difference in wellbeing measured using Warwick Edinburgh Mental Well-being scale (SWEMWBS) [45]
- Participant engagement with new activities

Economic evaluation measures will include:

- Difference in QALYs (incremental QALYs) between intervention and control at 6 months, with health related quality of life calculated using the SF-6D utility algorithm (derived from SF-12 data) [46]
- Difference in costs (incremental costs) of public sector resource between intervention and control at 6 months
- Cost utility (expressed in terms of Cost/QALY and Cost/year of sufficient capability)
- Difference in ICECAP-A scores between intervention and control at 6 months [47]

Process evaluation measures will include:

- Participant perceived collective efficacy measured using the Collective Efficacy in Networks Scale (CENS) [48] and social support using the SPA [49]
- Perceptions of loneliness measured using a modified version of the Brief Illness Perception questionnaire (modified B-IPQ) [50]

Intervention group only:

- Social network composition change measured using Genie social network mapping (intervention group only at 3-months)

Study Endpoints:

For peer review only - http://bmjopen.bmj.com/site/about/guidelines.xhtml

At 3- and 6-months after enrolment in to the study, patients will be invited to complete follow-up assessments. They may do this independently or with the assistance of the facilitator or a research team member (which may include online, on paper or over the phone). All follow-up assessments will be recorded no earlier than two weeks before the follow-up date and no later than six weeks after the follow-up date**.**

Measures:

See Table 1 for full details of study measures.

Table 1: Measures and schedule of observations within the PALS study

	Time point (month)		
**Measure**	Baseline	3 month follow-up	6 month follow-up
Socio-demographic measures	X		
**Patient self-report measures (both groups)**			
SF-12 Mental Health	X	X	X
SF-12 Physical Health	X	X	X
Loneliness (De Jong Scale)	X	X	X
Social isolation (Duke Social Support index)	X	X	X
Campaign to End Loneliness scale	X	X	X
Collective efficacy (CENS)	X	X	X
Social support (SPA)	X	X	X
Warwick Edinburgh Mental Well-being scale (SWEMWBS)	X	X	X
Perceptions of loneliness (modified B-IPQ)	X	X	X
Participant engagement with new activities	X	X	X
**Patient measures (network mapping, intervention group only)**			
Social network composition change at 3- and 6-month follow-up	X	X	X
**Economic measures**			

For peer review only

SF-6D	X	X	X
Capability wellbeing (ICECAP-A)	X	X	X
Health and social care use	X	X	X
**Process evaluation**			
Qualitative interviews with participants	X	X	X
Qualitative interviews with facilitators and stakeholders	X	X	X
Observations of facilitation	X		
Community staff observations of impact	X	X	X

For peer review only

Statistical analysis

All analyses will emphasise estimation and confidence intervals over hypothesis testing, and will be conducted as intention-to-treat. Missing data will be assumed to be missing at random, unless accounting for more than 10% of the sample; if missingness is above this rate, approaches for dealing with missing data (e.g. multiple imputation) will be discussed within the research team. Missingness will be reported for each arm and summaries of baseline characteristics of those lost to follow-up and those not will be used to judge potential sources of bias.

Baseline socio-demographic data will be summarised within randomised arms using appropriate descriptive measures; likewise, all outcome measures will be summarised by arm at each time-point. We will produce a forest plot of estimated effects for each outcome within each organisation to explore any variability in the impact of the intervention.

The primary analysis will involve a mixed effects model (pending the model meeting the associated assumptions) comparing groups on SF-12 at six months. The model will include a random intercept for facilitator and organisation, with participants clustered within facilitators clustered within organisation (hence a three-level model), and control for baseline SF-12. This analysis will be complemented by an analysis using the same framework but

For peer review only - http://bmjopen.bmj.com/site/about/guidelines.xhtml

with SF-12 as the outcome and a random coefficient for time, where repeated measurements are clustered within participants (hence a four-level model).

Non-response bias (i.e., where a particular group of participants are unavailable or refuse to participate) will be reduced by taking steps to increase the initial response rate and reduce drop-out over the course of the study.

Economic analysis

The primary analysis will be a cost-utility analysis (CUA) from a public sector perspective, with a primary outcome of cost/QALY at 6 months. Health related quality of life will be collected via SF-12 at baseline, 3 and 6 months, with utilities being derived by application of the SF-6D scoring algorithm [46]. In addition, scored values from the ICECAP-A [47, 51] will enable a secondary cost-utility analysis [47]. The use of ICECAP–A is planned to explore non-health attributes (specifically capabilities) that might be important to this population, thus allowing for a broader measurement of wellbeing than might be captured by SF-6D. While the comparative data collected on both measures may inform future studies in similar populations, it will also provide decision makers with richer information than would be obtained by a single generic HRQoL measure.

Intervention delivery resource use will be recorded on proformas designed to capture cost categories (e.g. trainer time, pay scale, intervention setting, facilitator travel costs). Additionally, at baseline, 3 and 6 months resource use will be collected directly from participants using a questionnaire designed to capture health care, social service and other public sector costs, as well as participant incurred costs (i.e. participant and carer costs). An exploratory analysis will use a societal perspective. The analysis of costs will therefore provide detail on the cost-shifts within sectors (e.g. health compared to social care) as well as providing decision makers with guidance on what, in the broadest sense, is optimal for

For peer review only - http://bmjopen.bmj.com/site/about/guidelines.xhtml

For peer review only

society [52]. All analysis will follow practice guidelines [53-55], including those related to public health and/or complex interventions specifically [56-58]. CUA will also allow for the construction of cost-effectiveness acceptability curves to assess whether the intervention is cost-effective at a range of payer thresholds [59]. Sub-group analysis will be carried out in order to inform policy makers' decision making with respect to the targeting of the intervention. Such sub-group analyses (for instance looking at intervention effects in different groups) will be planned prospectively, and quantitative analysis - foreseeably including mixed effects modelling to account for the clustered nature of the data [60] - will be set out as part of the statistical and health economic analysis plan. The economic evaluation will also be informed by the process evaluation terms of considering how the underlying mechanisms and contexts relate to resource use and cost areas [61]. Such an explanatory focus will be taken throughout the study, with a view to interpreting study results and assessing study generalisability.

Qualitative process evaluation and analysis

The qualitative process evaluation will combine complementary components to seek to provide an in-depth understanding of the factors that facilitate individual, environmental and organisational factors that inhibit or promote the engagement, workability, integration, sustainability and scalability of a social network intervention for addressing loneliness in open settings. The process evaluation will consider the pre-implementation contexts and processes, as well as observing use of the intervention in practice to understand the dynamics of implementation (including how the facilitation and other elements work) to consider implications for scale-up and sustainability for the participating organisations. Concepts from the Consolidated Framework for Implementation Research (CFIR) [62] will be used to guide the identification of factors promoting or inhibiting the routine incorporation and embeddedness of a facilitated social network intervention. The Non-adoption, Abandonment,

For peer review only - http://bmjopen.bmj.com/site/about/guidelines.xhtml

and Challenges to the Scale-up, Spread, and Sustainability (NASSS) framework will inform the evaluation of implementation because it has been designed to help predict and evaluate the success of a technology-supported health program, addressing concerns such as implementation, scale-up and sustainability [63]. An ethnographic approach making use of observations, interviews and documentary analysis will be used to capture the pre-implementation processes in order to explore the workability and integration of Genie in different community organisations. Following this, interviews will take place to explore engagement, sustainability and scalability. Participants will be sampled purposely based on circumstances of loneliness and socio-demographic factors; we will explore the experiences and meaning of loneliness. This will be combined with exploration of how individual circumstances shape engagement with different elements of the intervention, how change is enacted and embedded into people's everyday lives and how this involves other members of a person's network. We will describe the engagement and activities undertaken following the intervention including how links with new networks and resources are identified and made (navigation); how these are integrated (negotiation); and how new connections improve capacity to enact healthy behaviours, improve wellbeing or reduce isolation (collective efficacy). We will explore how facilitators felt about delivering Genie and how this might be adopted by their organisations as part of their practice. We will draw out new improvements and benefits specific to individual circumstances and existing use of health care services. Further interviews post-intervention will be conducted until 'saturation' (i.e. no significant new insights emerge).

ETHICS AND DISSEMINATION

Ethical approval

Ethical approval for the PALS study has been obtained from the South Central – Berkshire B ethics committee (reference: 15/SC/0245). All substantial amendments must be approved by

For peer review only - http://bmjopen.bmj.com/site/about/guidelines.xhtml

the University ethics committee and NHS ethics committee responsible for the trial, in additional to approval by HRA. Investigators are kept up to date with relevant changes via regular management group meetings.

Data monitoring

The Programme Steering Committee is responsible for ensuring programme adherence to the protocol, and adherence to the requirements of the Guidelines for Good Clinical Practice. The trial may be subject to inspection and audit by University of Southampton, under their remit as sponsor, the trial coordinating centre as the Sponsor's delegate and other regulatory bodies.

Dissemination

The findings from PALS will be disseminated widely through peer-reviewed publications, scientific conferences and workshops. In addition, we will aim to disseminate through multiple community pathways in collaboration with our partners and stakeholders (including local councils, NHS trusts and other local and national organisations) through interactive methods, such as targeted workshops, podcasts or blogs. If successful, we aim to aim to produce a user guide for applying Genie to loneliness and isolation.

For peer review only

Author contributions:

RB developed the initial idea for the study and obtained funding in collaboration with AR, SE, DC, IV, MCP, LY, RK, CB and the PALS study team. All authors have contributed to the protocol development. RB, TCB, JE and AR have led the trial preparations and development of training materials. IV has lead the development and modification of Genie. SE and DE led the statistical analysis planning, and KB and RK have led the health economics planning. JE, CB and AR have led the qualitative process evaluation planning. RB wrote the initial draft, all subsequent drafts were contributed to by all authors who have approved the final version.

Funding Statement: This protocol paper summarises independent research funded by the National Institute for Health Research (NIHR) under its Public Health Research programme (Grant Reference Number 16/08/41). The views expressed are those of the author(s) and not necessarily those of the NHS, the NIHR or the Department of Health.

Competing interests: None.

For peer review only - http://bmjopen.bmj.com/site/about/guidelines.xhtml

References

1. Yang, K. and C. Victor, *Age and loneliness in 25 European nations.* Ageing and Society, 2011. **31**(8): p. 1368-1388.
2. Seeman, T.E., *Health promoting effects of friends and family on health outcomes in older adults.* Am J Health Promot, 2000. **14**(6): p. 362-70.
3. Perissinotto, C.M., I. Stijacic Cenzer, and K.E. Covinsky, *Loneliness in older persons: a predictor of functional decline and death.* Arch Intern Med, 2012. **172**(14): p. 1078-83.
4. Victor, C.R. and K. Yang, *The prevalence of loneliness among adults: A case study of the United Kingdom.* Journal of Psychology 2012. **146**: p. 85-104.
5. Lasgaard, M., K. Friis, and M. Shevlin, *"Where are all the lonely people?" A population-based study of high-risk groups across the life span.* Soc Psychiatry Psychiatr Epidemiol, 2016. **51**(10): p. 1373-1384.
6. Victor, C.R., V. Burholt, and W. Martin, *Loneliness and Ethnic Minority Elders in Great Britain: An Exploratory Study.* Journal of Cross-Cultural Gerontology, 2012. **27**(1): p. 65-78.
7. ONS, *Loneliness - What characteristics and circumstances are associated with feeling lonely?*, O.o.N. Statistics, Editor. 2018.
8. Valtorta, N.K., et al., *Loneliness and social isolation as risk factors for coronary heart disease and stroke: systematic review and meta-analysis of longitudinal observational studies.* Heart, 2016.
9. Niedzwiedz, C.L., et al., *The relationship between wealth and loneliness among older people across Europe: Is social participation protective?* Prev Med, 2016. **91**: p. 24-31.
10. Conditions, D.L.T., *Long Term Conditions Compendium of Information* D.o. Health, Editor. 2012.
11. Coyle, C.E. and E. Dugan, *Social isolation, loneliness and health among older adults.* J Aging Health, 2012. **24**(8): p. 1346-63.
12. Luo, Y., et al., *Loneliness, health, and mortality in old age: a national longitudinal study.* Soc Sci Med, 2012. **74**(6): p. 907-14.
13. Cacioppo, J.T., et al., *Loneliness as a Specific Risk Factor for Depressive Symptoms: Cross-Sectional and Longitudinal Analyses.* Psychology and Aging

2006 **21**(1): p. 140 –151.
14. Theeke, L.A. and J. Mallow, *Loneliness and Quality of Life in Chronically Ill Rural Older Adults: Findings from a pilot study.* The American journal of nursing, 2013. **113**(9): p. 28-38.
15. Musich, S., et al., *Homebound older adults: Prevalence, characteristics, health care utilization and quality of care.* Geriatric Nursing, 2015. **36**(6): p. 445-450.
16. Shankar, A., et al., *Loneliness, social isolation, and behavioral and biological health indicators in older adults.* Health Psychol, 2011. **30**(4): p. 377-85.
17. Holt-Lunstad, J., T.B. Smith, and J.B. Layton, *Social Relationships and Mortality Risk: A Meta-analytic Review.* PLOS Medicine, 2010. **7**(7): p. e1000316.
18. Steptoe, A., et al., *Social isolation, loneliness, and all-cause mortality in older men and women.* Proceedings of the National Academy of Sciences, 2013. **110**(15): p. 5797-5801.
19. Holt-Lunstad, J., et al., *Loneliness and Social Isolation as Risk Factors for Mortality: A Meta-Analytic Review.* Perspectives on Psychological Science, 2015. **10**(2): p. 227-237.
20. Cohen, G.D., et al., *The impact of professionally conducted cultural programs on the physical health, mental health, and social functioning of older adults.* Gerontologist, 2006. **46**(6): p. 726-34.
21. Taube, E., et al., *Loneliness and health care consumption among older people.* Scand J Caring Sci, 2015. **29**(3): p. 435-43.

For peer review only - http://bmjopen.bmj.com/site/about/guidelines.xhtml

22. Qualter, P., et al., *Investigating Hypervigilance for Social Threat of Lonely Children.* Journal of Abnormal Child Psychology, 2013. **41**(2): p. 325-338.
23. Reblin, M. and B.N. Uchino, *Social and emotional support and its implication for health.* Curr Opin Psychiatry, 2008. **21**(2): p. 201-5.
24. Feng, X. and T. Astell-Burt, *What types of social interactions reduce the risk of psychological distress? Fixed effects longitudinal analysis of a cohort of 30,271 middle-to-older aged Australians.* Journal of Affective Disorders, 2016. **204**: p. 99-102.
25. NICE, *Mental wellbeing and independence for older people (QS137)*, D.o. Health, Editor. 2016, National Institute for Health and Care Excellence
26. Blakeman, T., et al., *Effect of information and telephone-guided access to community support for people with chronic kidney disease: randomised controlled trial.* PLoS One, 2014. **9**.
27. Kennedy, A., et al., *Implementing a social network intervention designed to enhance and diversify support for people with long-term conditions. A qualitative study.* Implementation Science, 2016. **11**(1): p. 27.
28. Reeves, D., et al., *The contribution of social networks to the health and self-management of patients with long-term conditions: a longitudinal study.* PLoS One, 2014. **9**.
29. Council, H.C., *Social isolation and loneliness in Hampshire: A health needs assessment*. 2016.
30. Ware, J., Jr., M. Kosinski, and S.D. Keller, *A 12-Item Short-Form Health Survey: construction of scales and preliminary tests of reliability and validity.* Med Care, 1996. **34**(3): p. 220-33.
31. Dickens, A.P., et al., *An evaluation of the effectiveness of a community mentoring service for socially isolated older people: a controlled trial.* BMC Public Health, 2011. **11**(1): p. 218.
32. Parker, S.L., et al., *Assessment of the minimum clinically important difference in pain, disability, and quality of life after anterior cervical discectomy and fusion: clinical article.* J Neurosurg Spine, 2013. **18**(2): p. 154-60.
33. Elley, C.R., et al., *Intraclass correlation coefficients from three cluster randomised controlled trials in primary and residential health care.* Aust N Z J Public Health, 2005. **29**(5): p. 461-7.
34. Littenberg, B. and C.D. MacLean, *Intra-cluster correlation coefficients in adults with diabetes in primary care practices: the vermont diabetes information system field survey.* BMC Med Res Methodol, 2006. **6**.
35. Blickem, C., et al., *The clinical and cost-effectiveness of the BRinging information and guided help together (BRIGHT) intervention for the self-management support of people with stage 3 chronic kidney disease in primary care: study protocol for a randomized controlled trial.* Trials, 2013. **14**.
36. Rogers, A., et al., *Social networks, work and network-based resources for the management of long-term conditions: a framework and study protocol for developing self-care support.* Implementation Sci, 2011. **6**.
37. Vassilev, I., et al., *Social networks, social capital and chronic illness self-management: a realist review.* Chronic Illness, 2011. **7**.
38. Vassilev, I., et al., *Social networks, the 'work'and work force of chronic illness self-management: a survey analysis of personal communities.* PloS One, 2013. **8**.
39. Thoits, P., *Mechanisms linking social ties and support to physical and mental health.* J Health Hum Behav, 2011. **52**.
40. Blickem, C., et al., *Linking people with long-term health conditions to healthy community activities: development of PLANS (patient-Led assessment for network support).* Health Expect, 2013. **16**.
41. Blickem, C., et al., *Aligning everyday life priorities with people's self-management support networks: an exploration of the work and implementation of a needs-led telephone support system.* BMC Health Services Research, 2014. **14**(1): p. 262.
42. Gierveld, J.D.J. and T.V. Tilburg, *A 6-Item Scale for Overall, Emotional, and Social Loneliness: Confirmatory Tests on Survey Data.* Research on Aging, 2006. **28**(5): p. 582-598.
43. loneliness, C.a., *MEASURING YOUR IMPACT ON LONELINESS IN LATER LIFE*.

For peer review only - http://bmjopen.bmj.com/site/about/guidelines.xhtml

44. Koenig, H.G., et al., *Abbreviating the Duke Social Support Index for use in chronically ill elderly individuals.* Psychosomatics, 1993. **34**(1): p. 61-9.
45. NHS Health Scotland, U.o.W.a.U.o.E., *Short Warwick Edinburgh Mental Well-Being Scale (SWEMWBS).* 2008.
46. Brazier, J.E. and J. Roberts, *The estimation of a preference-based measure of health from the SF-12.* Med Care, 2004. **42**(9): p. 851-9.
47. Flynn, T.N., et al., *Scoring the Icecap-a capability instrument. Estimation of a UK general population tariff.* Health Econ, 2015. **24**(3): p. 258-69.
48. Band, R., et al., *Development of a measure of Collective Efficacy within personal networks: a complement to self-efficacy in self-management support? .* Patient Educ Couns, In submission
49. Cutrona, C.E. and D.W. Russell, *The provisions of social relationships and adaptation to stress.* Advances in personal relationships, 1987. **1**(1): p. 37-67.
50. Broadbent, E., et al., *The brief illness perception questionnaire.* J Psychosom Res, 2006. **60**(6): p. 631-7.
51. Al-Janabi, H., T. N Flynn, and J. Coast, *Development of a self-report measure of capability wellbeing for adults: the ICECAP-A.* Quality of Life Research, 2012. **21**(1): p. 167-176.
52. Jonsson, B., *Ten arguments for a societal perspective in the economic evaluation of medical innovations.* Eur J Health Econ, 2009. **10**(4): p. 357-9.
53. NICE, *Guide to the Methods of Technology appraisal*. 2013
54. Gold, M.R., *Cost-effectiveness in health and medicine*. Vol. xxiii. 1996, New York: Oxford University Press.
55. Glick, H., *Economic evaluation in clinical trials*. Vol. x,. 2007, New York: Oxford University Press.
56. Weatherly, H., et al., *Methods for assessing the cost-effectiveness of public health interventions.* key challenges and recommendations, 2009. **93**(2-3): p. 85-92.
57. Craig, P., et al., *Developing and evaluating complex interventions: The new Medical Research Council guidance.* International Journal of Nursing Studies, 2013. **50**(5): p. 587-592.
58. Payne, K. and A. Thompson, *Economic evaluations of complex interventions.* , in *Complex Interventions in Health: An Overview of Research Methods*, D.A. Richards and I.R. Hallberg, Editors. 2015, Routledge: Abingdon, UK. p. 248–257.
59. Fenwick, E., B.J. O'Brien, and A. Briggs, *Cost-effectiveness acceptability curves--facts, fallacies and frequently asked questions.* Health Econ, 2004. **13**(5): p. 405-15.
60. Sculpher, M.J., et al., *Generalisability in economic evaluation studies in healthcare: a review and case studies.* Health Technol Assess, 2004. **8**(49): p. iii-iv, 1-192.
61. Anderson, R. and R. Hardwick, *Realism and resources: Towards more explanatory economic evaluation.* Evaluation (Lond), 2016. **22**(3): p. 323-341.
62. Damschroder, L.J., et al., *Fostering implementation of health services research findings into practice: a consolidated framework for advancing implementation science.* Implementation Science, 2009. **4**(1): p. 50.
63. Greenhalgh, T., et al., *Beyond Adoption: A New Framework for Theorizing and Evaluating Nonadoption, Abandonment, and Challenges to the Scale-Up, Spread, and Sustainability of Health and Care Technologies.* J Med Internet Res, 2017. **19**(11): p. e367.

For peer review only

For peer review only - http://bmjopen.bmj.com/site/about/guidelines.xhtml

SPIRIT

STANDARD PROTOCOL ITEMS: RECOMMENDATIONS FOR INTERVENTIONAL TRIALS

SPIRIT 2013 Checklist: Recommended items to address in a clinical trial protocol and related documents*

Section/item	Item No	Description	Addressed on page number
**Administrative information**			
Title	1	Descriptive title identifying the study design, population, interventions, and, if applicable, trial acronym	1
Trial registration	2a	Trial identifier and registry name. If not yet registered, name of intended registry	3
	2b	All items from the World Health Organization Trial Registration Data Set	______
Protocol version	3	Date and version identifier	1
Funding	4	Sources and types of financial, material, and other support	20
Roles and responsibilities	5a	Names, affiliations, and roles of protocol contributors	1
	5b	Name and contact information for the trial sponsor	______
	5c	Role of study sponsor and funders, if any, in study design; collection, management, analysis, and interpretation of data; writing of the report; and the decision to submit the report for publication, including whether they will have ultimate authority over any of these activities	20
	5d	Composition, roles, and responsibilities of the coordinating centre, steering committee, endpoint adjudication committee, data management team, and other individuals or groups overseeing the trial, if applicable (see Item 21a for data monitoring committee)	______

For peer review only

**Introduction**			
Background and rationale	6a	Description of research question and justification for undertaking the trial, including summary of relevant studies (published and unpublished) examining benefits and harms for each intervention	5/6
	6b	Explanation for choice of comparators	12
Objectives	7	Specific objectives or hypotheses	6
Trial design	8	Description of trial design including type of trial (eg, parallel group, crossover, factorial, single group), allocation ratio, and framework (eg, superiority, equivalence, noninferiority, exploratory)	6/7
**Methods: Participants, interventions, and outcomes**			
Study setting	9	Description of study settings (eg, community clinic, academic hospital) and list of countries where data will be collected. Reference to where list of study sites can be obtained	6/7
Eligibility criteria	10	Inclusion and exclusion criteria for participants. If applicable, eligibility criteria for study centres and individuals who will perform the interventions (eg, surgeons, psychotherapists)	7/8
Interventions	11a	Interventions for each group with sufficient detail to allow replication, including how and when they will be administered	10/11
	11b	Criteria for discontinuing or modifying allocated interventions for a given trial participant (eg, drug dose change in response to harms, participant request, or improving/worsening disease)	______________
	11c	Strategies to improve adherence to intervention protocols, and any procedures for monitoring adherence (eg, drug tablet return, laboratory tests)	______________
	11d	Relevant concomitant care and interventions that are permitted or prohibited during the trial	______________
Outcomes	12	Primary, secondary, and other outcomes, including the specific measurement variable (eg, systolic blood pressure), analysis metric (eg, change from baseline, final value, time to event), method of aggregation (eg, median, proportion), and time point for each outcome. Explanation of the clinical relevance of chosen efficacy and harm outcomes is strongly recommended	12-14
Participant timeline	13	Time schedule of enrolment, interventions (including any run-ins and washouts), assessments, and visits for participants. A schematic diagram is highly recommended (see Figure)	8/9

For peer review only - http://bmjopen.bmj.com/site/about/guidelines.xhtml

Sample size	14	Estimated number of participants needed to achieve study objectives and how it was determined, including clinical and statistical assumptions supporting any sample size calculations	9
Recruitment	15	Strategies for achieving adequate participant enrolment to reach target sample size	7

Methods: Assignment of interventions (for controlled trials)

Allocation:

Sequence generation	16a	Method of generating the allocation sequence (eg, computer-generated random numbers), and list of any factors for stratification. To reduce predictability of a random sequence, details of any planned restriction (eg, blocking) should be provided in a separate document that is unavailable to those who enrol participants or assign interventions	8
Allocation concealment mechanism	16b	Mechanism of implementing the allocation sequence (eg, central telephone; sequentially numbered, opaque, sealed envelopes), describing any steps to conceal the sequence until interventions are assigned	8
Implementation	16c	Who will generate the allocation sequence, who will enrol participants, and who will assign participants to interventions	8
Blinding (masking)	17a	Who will be blinded after assignment to interventions (eg, trial participants, care providers, outcome assessors, data analysts), and how	______________
	17b	If blinded, circumstances under which unblinding is permissible, and procedure for revealing a participant's allocated intervention during the trial	______________

Methods: Data collection, management, and analysis

Data collection methods	18a	Plans for assessment and collection of outcome, baseline, and other trial data, including any related processes to promote data quality (eg, duplicate measurements, training of assessors) and a description of study instruments (eg, questionnaires, laboratory tests) along with their reliability and validity, if known. Reference to where data collection forms can be found, if not in the protocol	15
	18b	Plans to promote participant retention and complete follow-up, including list of any outcome data to be collected for participants who discontinue or deviate from intervention protocols	______________

Data management	19	Plans for data entry, coding, security, and storage, including any related processes to promote data quality (eg, double data entry; range checks for data values). Reference to where details of data management procedures can be found, if not in the protocol	
Statistical methods	20a	Statistical methods for analysing primary and secondary outcomes. Reference to where other details of the statistical analysis plan can be found, if not in the protocol	15
	20b	Methods for any additional analyses (eg, subgroup and adjusted analyses)	
	20c	Definition of analysis population relating to protocol non-adherence (eg, as randomised analysis), and any statistical methods to handle missing data (eg, multiple imputation)	
**Methods: Monitoring**			
Data monitoring	21a	Composition of data monitoring committee (DMC); summary of its role and reporting structure; statement of whether it is independent from the sponsor and competing interests; and reference to where further details about its charter can be found, if not in the protocol. Alternatively, an explanation of why a DMC is not needed	
	21b	Description of any interim analyses and stopping guidelines, including who will have access to these interim results and make the final decision to terminate the trial	
Harms	22	Plans for collecting, assessing, reporting, and managing solicited and spontaneously reported adverse events and other unintended effects of trial interventions or trial conduct	
Auditing	23	Frequency and procedures for auditing trial conduct, if any, and whether the process will be independent from investigators and the sponsor	
**Ethics and dissemination**			
Research ethics approval	24	Plans for seeking research ethics committee/institutional review board (REC/IRB) approval	18
Protocol amendments	25	Plans for communicating important protocol modifications (eg, changes to eligibility criteria, outcomes, analyses) to relevant parties (eg, investigators, REC/IRBs, trial participants, trial registries, journals, regulators)	

Consent or assent	26a	Who will obtain informed consent or assent from potential trial participants or authorised surrogates, and how (see Item 32)	________
	26b	Additional consent provisions for collection and use of participant data and biological specimens in ancillary studies, if applicable	________
Confidentiality	27	How personal information about potential and enrolled participants will be collected, shared, and maintained in order to protect confidentiality before, during, and after the trial	________
Declaration of interests	28	Financial and other competing interests for principal investigators for the overall trial and each study site	20
Access to data	29	Statement of who will have access to the final trial dataset, and disclosure of contractual agreements that limit such access for investigators	________
Ancillary and post-trial care	30	Provisions, if any, for ancillary and post-trial care, and for compensation to those who suffer harm from trial participation	________
Dissemination policy	31a	Plans for investigators and sponsor to communicate trial results to participants, healthcare professionals, the public, and other relevant groups (eg, via publication, reporting in results databases, or other data sharing arrangements), including any publication restrictions	19
	31b	Authorship eligibility guidelines and any intended use of professional writers	________
	31c	Plans, if any, for granting public access to the full protocol, participant-level dataset, and statistical code	________
**Appendices**			
Informed consent materials	32	Model consent form and other related documentation given to participants and authorised surrogates	________
Biological specimens	33	Plans for collection, laboratory evaluation, and storage of biological specimens for genetic or molecular analysis in the current trial and for future use in ancillary studies, if applicable	________

*It is strongly recommended that this checklist be read in conjunction with the SPIRIT 2013 Explanation & Elaboration for important clarification on the items. Amendments to the protocol should be tracked and dated. The SPIRIT checklist is copyrighted by the SPIRIT Group under the Creative Commons "Attribution-NonCommercial-NoDerivs 3.0 Unported" license.

For peer review only - http://bmjopen.bmj.com/site/about/guidelines.xhtml

CONSORT 2010 checklist of information to include when reporting a randomised trial*

Section/Topic	Item No	Checklist item	Reported on page No
**Title and abstract**			
	1a	Identification as a randomised trial in the title	1
	1b	Structured summary of trial design, methods, results, and conclusions (for specific guidance see CONSORT for abstracts)	2
**Introduction**			
Background and objectives	2a	Scientific background and explanation of rationale	4-5
	2b	Specific objectives or hypotheses	6
**Methods**			
Trial design	3a	Description of trial design (such as parallel, factorial) including allocation ratio	6-7
	3b	Important changes to methods after trial commencement (such as eligibility criteria), with reasons	n/a
Participants	4a	Eligibility criteria for participants	7-8
	4b	Settings and locations where the data were collected	6-7
Interventions	5	The interventions for each group with sufficient details to allow replication, including how and when they were actually administered	10-12
Outcomes	6a	Completely defined pre-specified primary and secondary outcome measures, including how and when they were assessed	12-13
	6b	Any changes to trial outcomes after the trial commenced, with reasons	n/a
Sample size	7a	How sample size was determined	9
	7b	When applicable, explanation of any interim analyses and stopping guidelines	n/a
Randomisation:			
Sequence generation	8a	Method used to generate the random allocation sequence	8
	8b	Type of randomisation; details of any restriction (such as blocking and block size)	8
Allocation concealment mechanism	9	Mechanism used to implement the random allocation sequence (such as sequentially numbered containers), describing any steps taken to conceal the sequence until interventions were assigned	8
Implementation	10	Who generated the random allocation sequence, who enrolled participants, and who assigned participants to interventions	8
Blinding	11a	If done, who was blinded after assignment to interventions (for example, participants, care providers, those	n/a

For peer review only

		assessing outcomes) and how	
	11b	If relevant, description of the similarity of interventions	n/a
Statistical methods	12a	Statistical methods used to compare groups for primary and secondary outcomes	15
	12b	Methods for additional analyses, such as subgroup analyses and adjusted analyses	16
**Results**			
Participant flow (a diagram is strongly recommended)	13a	For each group, the numbers of participants who were randomly assigned, received intended treatment, and were analysed for the primary outcome	n/a
	13b	For each group, losses and exclusions after randomisation, together with reasons	n/a
Recruitment	14a	Dates defining the periods of recruitment and follow-up	9
	14b	Why the trial ended or was stopped	n/a
Baseline data	15	A table showing baseline demographic and clinical characteristics for each group	n/a
Numbers analysed	16	For each group, number of participants (denominator) included in each analysis and whether the analysis was by original assigned groups	n/a
Outcomes and estimation	17a	For each primary and secondary outcome, results for each group, and the estimated effect size and its precision (such as 95% confidence interval)	n/a
	17b	For binary outcomes, presentation of both absolute and relative effect sizes is recommended	n/a
Ancillary analyses	18	Results of any other analyses performed, including subgroup analyses and adjusted analyses, distinguishing pre-specified from exploratory	n/a
Harms	19	All important harms or unintended effects in each group (for specific guidance see CONSORT for harms)	n/a
**Discussion**			
Limitations	20	Trial limitations, addressing sources of potential bias, imprecision, and, if relevant, multiplicity of analyses	n/a
Generalisability	21	Generalisability (external validity, applicability) of the trial findings	n/a
Interpretation	22	Interpretation consistent with results, balancing benefits and harms, and considering other relevant evidence	n/a
**Other information**			
Registration	23	Registration number and name of trial registry	3
Protocol	24	Where the full trial protocol can be accessed, if available	n/a
Funding	25	Sources of funding and other support (such as supply of drugs), role of funders	20

*We strongly recommend reading this statement in conjunction with the CONSORT 2010 Explanation and Elaboration for important clarifications on all the items. If relevant, we also recommend reading CONSORT extensions for cluster randomised trials, non-inferiority and equivalence trials, non-pharmacological treatments, herbal interventions, and pragmatic trials. Additional extensions are forthcoming: for those and for up to date references relevant to this checklist, see www.consort-statement.org.

For peer review only - http://bmjopen.bmj.com/site/about/guidelines.xhtml

BMJ Open

Study protocol for 'The Project About Loneliness and Social networks (PALS)': a pragmatic, randomised trial comparing a facilitated social network intervention (Genie) with a wait-list control for lonely and socially-isolated people

Journal:	*BMJ Open*
Manuscript ID	bmjopen-2018-028718.R1
Article Type:	Protocol
Date Submitted by the Author:	13-Mar-2019
Complete List of Authors:	Band, Rebecca; University of Southampton, Psychology ; University of Southampton, Health Sciences<br>Ewings, Sean; University of Southampton, Health Sciences<br>Cheetham-Blake, Tara; University of Southampton, Faculty of Health Sciences<br>Ellis, Jaimie; University of Southampton, Health Sciences<br>Breheny, Katie; University of Bristol, Population Health Sciences<br>Vassilev, Ivaylo; University of Southampton, Health Sciences<br>Portillo , Mari Carmen; University of Southampton, Health Sciences<br>Yardley, Lucy; University of Southampton, Academic Unit of Psychology; University of Bristol, School of Psychological Science<br>Blickem, C; Liverpool John Moores University, Public Health<br>Kandiyali, Rebecca; University of Bristol School of Social and Community Medicine, Centre for Child and Adolescent Health<br>Culliford, David; University of Southampton, Faculty of Medicine<br>Rogers, Anne; University of Southampton, Faculty of Health Sciences
**Primary Subject Heading**:	Public health
Secondary Subject Heading:	Research methods
Keywords:	Protocol, Randomised Controlled Trial, Social networks, Social isolation, Loneliness

SCHOLARONE™
Manuscripts

Study protocol for 'The Project About Loneliness and Social networks (PALS)': a pragmatic, randomised trial comparing a facilitated social network intervention (Genie) with a wait-list control for lonely and socially-isolated people

Protocol version: 1

This corresponds with the study protocol version 4.

Rebecca Band[1,2], Sean Ewings[1], Tara Cheetham-Blake[1], Jaimie Ellis[1], Katie Breheny[3], Ivaylo Vassilev[1], Mari Carmen Portillo[1], Lucy Yardley[2, 4], Christian Blickem[5], Rebecca Kandiyali[3], David Culliford[1], Anne Rogers[1]

[1] NIHR CLAHRC Wessex Health Sciences, University of Southampton, Southampton, UK

[2] Academic unit of psychology, University of Southampton, Southampton, UK

[3] Population Health Sciences, Bristol Medical School, University of Bristol, UK

[4] School of Psychological Science, University of Bristol, UK

[5] Public Health Institute, Faculty of Health, Education and Community, Liverpool John Moores University, Liverpool, UK

Corresponding author: Rebecca Band, Highfield Campus, University of Southampton, Southampton. SO17 1BJ. Email: r.j.band@soton.ac.uk

Keywords: Loneliness, Social isolation, Social networks, Randomised Controlled Trial,

Protocol

Word count: 4499

For peer review only - http://bmjopen.bmj.com/site/about/guidelines.xhtml

Abstract:

Introduction:

Loneliness and social isolation have been identified as significant public health concerns, but improving relationships and increasing social participation may improve health outcomes and quality of life. The aim of the PALS study is to assess the effectiveness and cost-effectiveness of a guided social network intervention within a community setting among individuals experiencing loneliness and isolation and to understand implementation of Genie in the context of different organisations.

Methods and analysis:

The PALS trial will be a pragmatic, randomised controlled trial comparing participants receiving the Genie intervention to a wait-list control group. Eligible participants will be recruited from organisations working within a community setting: any adult identified as socially isolated or at-risk of loneliness and living in the community will be eligible. Genie will be delivered by trained facilitators recruited from community organisations. The primary outcome will be the difference in the SF-12 Mental Health composite scale score at 6-month follow-up between the intervention and control group using a mixed effects model (accounting for clustering within facilitators and organisation). Secondary outcomes will be loneliness; social isolation; wellbeing; physical health and engagement with new activities. The economic evaluation will use a cost-utility approach, and adopt a public sector perspective to include health-related resource use and costs incurred by other public services. Exploratory analysis will use a societal perspective, and explore broader measures of benefit (capability wellbeing). A qualitative process evaluation will explore organisational and environmental arrangements, as well as stakeholder and participant experiences of the study

For peer review only - http://bmjopen.bmj.com/site/about/guidelines.xhtml

to understand the factors likely to influence future sustainability, implementation and scalability of using a social network intervention within this context.

Ethics and dissemination:

This study has received NHS ethical approval (REC reference: 18/SC/0245). The findings from PALS will be disseminated widely through peer-reviewed publications, conferences and workshops in collaboration with our community partners.

Trial registration number: ISRCTN 19193075

300/300 words

Strengths and limitations

- This study will evaluate an existing social network intervention (Genie) in the context of loneliness and social isolation
- The PALS study consists of a pragmatic RCT implemented in conjunction with community-based stakeholders in a community setting in two areas of the UK
- The process evaluation and analysis has been designed to understand the factors influencing the implementation and scalability of social network interventions in this context

For peer review only

INTRODUCTION

Social isolation is considered to be an objective lack of social connections, contact or participation, while loneliness is a subjective psychological state where there is a discrepancy between desired and perceived levels of support or connectedness [1, 2]. The prevalence rates of loneliness and isolation vary [3], however it is estimated to affect about 30% of the adult population in the UK [4]. Specific at-risk groups, such as the elderly, minority communities, and those with long-term mental or physical health conditions are significantly more isolated than those in good health [3, 5, 6]. The Office of National Statistics (ONS) recently identified three profiles of individuals who are 'at-risk'; these suggest different factors may be important in the experience of loneliness at different points across the life-course [7].

The problem: health implications of loneliness and social isolation

The impact of loneliness and isolation on well-being and the associated health risks have been identified as a significant public health concern [8, 9] exacerbated by the prevalence of long-term conditions and advancing age [10]. Both loneliness and social isolation are associated with poor physical and mental health outcomes [11-13], reduced quality of life [14, 15] and is linked to poorer physiological outcomes such as raised blood pressure and increased health-risk behaviours (e.g. sedentary behaviour) [16]. Their impact on mortality is estimated to exceed that of traditional risk factors such as obesity and cigarette smoking, with a 50% higher risk compared with socially-integrated participants [17-19]. There are also significant costs associated with raised demand and use of health services, and loneliness is associated with increased GP appointments, emergency hospital admittance and premature social care use [20-22].

Social relationships and preventing or reducing loneliness and social isolation

For peer review only

For peer review only - http://bmjopen.bmj.com/site/about/guidelines.xhtml

Although the determinants of loneliness and isolation are varied, social and emotional support from others is likely to be protective [23], with emerging evidence suggesting that improving the quality of interpersonal relationships and participation in social activities may be key to tackling the impact of loneliness [9]. Evidence has indicated that increasing social interactions and the number of people who can be relied on is associated with reduced levels of distress [24], whilst connecting with community resources can help protect against loneliness for those who are most at risk [9, 25]. Furthermore, there is evidence that social network interventions can significantly improve health outcomes, quality of life and increase the take-up of new activities [26, 27]. A diverse and supportive network has been shown to reduce health service costs [28]. A recent NICE quality standard recommends the navigation of older vulnerable people to community activities as a means of preventing loneliness in this group [25].

Rationale and risk-benefits for the current trial

In line with this evidence, there is a logical argument for introducing an effective social network intervention outside of formal healthcare settings to connect people who are experiencing loneliness to others within their communities [25]. Creative engagement with non-traditional informal providers of wellness management (such as through accessing locally available community groups) offers an alternative opportunity to address health and social needs. We envisage that the study will offset any burden through providing wider benefit to organisations; firstly through staff development and training integrating the intervention into practice, and, secondly, by providing a resource and alternative referral pathway for individuals who they have identified at risk of isolation or loneliness (potentially extending beyond the life of the study). A series of nestled qualitative process studies will

For peer review only - http://bmjopen.bmj.com/site/about/guidelines.xhtml

examine the context, practices and processes relating to implementing the intervention within the community context, and an economic evaluation to assess whether this is cost-effective.

Study aims and research questions

The aim of the PALS study is to assess the feasibility, acceptability, effectiveness and cost-effectiveness of a facilitated social network intervention compared to a wait-list control within a community setting among at-risk populations, and to understand the implementation in the context of different organisations who work in this environment. The Genie (Generating Engagement in Network Involvement) intervention is an online, facilitated, social networking tool designed to develop opportunities for social involvement.

Primary objectives

- To determine the effect of Genie compared to usual care on mental health (SF-12 composite scale score) at three and six months.

Secondary objectives

- To determine the effect of Genie compared to usual care on loneliness, social isolation, physical health, and engagement with new activities at three and six months.
- To establish whether the use of Genie within a community setting is cost-effective.

Process analysis objectives

- To assess the acceptability and feasibility of running the study based on recruitment and retention during an internal pilot phase.
- To explore the experiences of using Genie, how the intervention impacts on loneliness and isolation, and the mechanisms by which participants enact change.

For peer review only - http://bmjopen.bmj.com/site/about/guidelines.xhtml

- To explore contextual environmental and organisational factors that inhibit or promote the integration, sustainability and scalability of Genie for addressing loneliness in local and organisational settings.

METHODS AND ANALYSIS

Study design and setting

We will conduct a pragmatic, randomised controlled trial comparing participants receiving the facilitated social network Genie intervention to a wait-list control group; randomisation will be at individual and/or cluster (facilitator) level (see Randomisation section). We will work closely with community partners two localities (centred around Southampton and Liverpool) in identifying participants and delivering the intervention, as well as informing our understanding of the challenges and environmental factors associated with implementation. Partners may include any group or organisation that has the potential to identify or access at-risk individuals.

Study participants

Identification

We will use a multi-stranded recruitment strategy to reflect the diversity of individuals who are living with loneliness or in isolation. This will be facilitated by collaborating community organisations to ensure that we are able to identify and access those most at-risk. Potential participants will be identified in the manner that best operates within existing working practices for each organisation (which will be different for each organisation/ collaborator). This is necessary to explore the integration and scalability of Genie in local and organisational settings. Potential participants will be invited by the organisation; this may be by letter or during routine visits, appointments, or in line with the usual working practices of the partner organisation. This may include (but is not limited to) new referrals, waiting lists,

For peer review only - http://bmjopen.bmj.com/site/about/guidelines.xhtml

or opportunistic contacts during routine work of partner organisation. All eligible participants will be given a research pack including an invitation letter, participant information sheet, and freepost reply slip to return should they wish to take part in the trial.

Eligibility criteria

We will recruit any adult (aged 18 or over) who is identified as being isolated or at risk of loneliness. We define a socially isolated person as one for whom there is an "absence of social contacts or community involvement, or lack of access to services" in line with the definition used by Hampshire County Council [29].

Exclusion Criteria

Exclusion criteria will include participants who are:

- currently hospitalised (i.e. not self-managing within a community setting)
- , those in the end stages of life or any condition which impacts upon ability to take part
- those lacking sufficient capacity
- and those having previously used the Genie intervention.

Eligibility will be assessed by the community partners and confirmed by the research team in all cases. Randomisation

The randomisation process is partly determined by the structure of each organisation and is designed to ensure that a) the risk of contamination across study arms is minimised, and b) allocation concealment is maintained. Facilitators will be randomised (1:1) to either the intervention or control arm where possible, and participants also randomly allocated (1:1) to the corresponding arm via an independent process within each organisation. In this case, facilitator randomisation will be stratified by organisation with blocks of two (i.e., one

For peer review only - http://bmjopen.bmj.com/site/about/guidelines.xhtml

facilitator will be randomised to the intervention arm and one to the control arm) and conducted by the trial statistician (SE). Where there are practical constraints on facilitators who work within a specific locality (i.e. geographical, services or otherwise), the facilitator will be randomised (1:1) to either the intervention or control arm but participants within the locality will not be randomised. In instances where facilitators do not have an ongoing relationship with potential participants, and none of the above constraints apply, only participants will be randomised (1:1). Randomisation models are outlined in Table 1. Blocking will occur in all cases. Randomisation sequences will be computer-generated. The sequences will be stored in sealed, opaque, numbered envelopes so that researchers are blinded to participant allocation. For facilitators within an organisation, assignment to intervention or control will happen simultaneously once they have agreed to take part in the study.

Table 1: The factors affecting the recruitment and randomisation process

		Contact between participant and facilitator	
		**Ongoing**	**One-off contact (at facilitation)**
Participant recruitment	**Area/ location not restricted**	MODEL B<br>• Train intervention facilitators only (ideally)<br>• Randomise facilitators<br>• Participants also randomised<br>• Recruitment by facilitator possible (assuming no prior connection to participant)	MODEL A<br>• Train intervention facilitators only (ideally)<br>• Participants only randomised<br>• Recruitment by facilitator possible
	**Within a specific geographical (or other pre-specified) area**	MODEL C<br>• Train intervention facilitators only (ideally)<br>• Randomise facilitators only<br>• Participants within each area allocated to facilitator (not randomised)<br>• Recruitment by non-facilitator	MODEL D<br>• Can train all facilitators<br>• Randomise facilitators only<br>• Participants within each area allocated to facilitator (not randomised)<br>• Recruitment by non-facilitator

For peer review only

For peer review only - http://bmjopen.bmj.com/site/about/guidelines.xhtml

Participant flow through the study

Written informed consent will be collected from all participants and baseline data collected with a research team member (online or on paper, dependent on the participant preferences). Allocation will occur once the baseline assessment has been completed. Participants who are allocated to the intervention condition will be given access to the Genie intervention within two weeks of the baseline appointment; this process will be guided by the facilitator at a location to suit them (i.e. at home or in the community). At 3 and 6 months after enrolment into the study, participants will be invited to complete follow-up assessments. All follow-up assessments will be recorded no earlier than two weeks before the follow-up date and no later than six weeks after the follow-up date. Each participant will be sent a £10 high street gift voucher with the 6-month follow-up questionnaire. Individuals allocated to the control group will be offered access to Genie with the facilitator after the have completed their 6-month follow-up assessment. Participant flow is outlined in Figure 1.

Sample size consideration

The sample size calculation is based on the primary analysis of the comparison of intervention and control arms on SF-12 Mental Health composite scale (MCS) score at six months [30], and accounts for possible intra-cluster correlation (ICC) within facilitators. The MCS compares an individual score to an age group mean score; a negative score reflects poorer health. Previous studies (albeit in different populations) have suggested that differences of 3 and 4.7 points on the SF-12 would be clinically meaningful [31, 32]. We have based the current sample size on being able to detect a difference of 4 points. Based on a previous study in socially-isolated older people [33], we estimate the standard deviation of the outcome to be 10.4 (using a pooled estimate of baseline scores). Choosing 80% power and a type I error rate of 5%, an individually-randomised study would require 216 people

For peer review only - http://bmjopen.bmj.com/site/about/guidelines.xhtml

(108 per arm). Regarding clustering, previous studies have generally shown low ICCs for mental health scores from SF-12 and SF-36 (0.032 and below, albeit for different populations and clustering within GP practices) [33, 34]; we use an ICC of 0.05 here. Based on discussions with participating organisations, it was agreed that 12 participants per facilitator was suitable; this results in a design effect of 1.55 and an adjusted sample size of 335 people. Assuming 15% drop-out [35], we require 394 participants in total (197 per arm). This requires 33 facilitators; we will increase this to 36 facilitators to account for potential drop-out of facilitators.

The facilitated Social network Intervention

The intervention process is introduced initially via a guided discussion with a trained facilitator, takes 30 to 40 minutes to deliver and has three stages: social network mapping, tailoring of preferences, linking users to valued resources and activities. By design, Genie (https://pals.genie-net.org/eng/), can be applied to varied user groups [27]. It is based on evidence of social network properties and types, mechanisms and work relating to managing health and wellness [36-39].Previous testing of the principles has shown that it is both appropriate and acceptable to implement for individuals with a long- term condition [26-28].

Facilitators

Guided facilitation is an important element of the guided process to using the tool. Facilitators do not need a specific in-depth theoretical knowledge: instead, the local knowledge of facilitators is important and adds to the value of the intervention. However, the interpersonal skills of the facilitator are vital for the success of engagement through promoting a collaborative solution, and engaging participant focus, motivation and reflection on social network composition and promoting new community engagement [27]. Facilitators receive a minimum of a half-day training from the research team, which may be refreshed

For peer review only - http://bmjopen.bmj.com/site/about/guidelines.xhtml

over the course of the study. This will include a background to, demonstration of, and practical pair-working exercises using video guides around the facilitation process. Research methods training and discussion around loneliness and isolation are also addressed. The research team will provide ongoing support to monitor fidelity to the intervention deployment and address issues arising regarding complex cases (or facilitator difficulties and distress).

Social network mapping

Facilitators guide participants to create a visual map of their current support network, using a concentric circles method [27]. The concentric circles process provides insight into the user's current situation regarding social support; who they view as important in their daily lives (this may include family members, friends, acquaintances, healthcare professionals, local groups and pets); and then to reflect on renegotiating existing roles and responsibilities, and further map people and groups who could provide extended support [26-28]. This process, when guided by the facilitator, helps the participant to realign thinking about their relationships (and conceptualise themselves within a network of support), explore family dynamics and recognise 'weak ties' (i.e. social acquaintances) that already exist in their network [27]. It also offers the opportunity to begin discussions about how support may be extended within the network.

Linking individuals with preferences and valued local and online activities and resources

The next step involves facilitating access to local resources based on personal preferences, and acceptability to encourage engagement with personal choices, through a set of 13 questions [40]. The questions generate a set of preferred local and online resources (linked to a pre-created database of categorised local organisations and resources). The facilitated discussion of preferences is linked to available and accepted potential support from people in a person's network. Personalised results are presented in a user-friendly way aided by Google

For peer review only - http://bmjopen.bmj.com/site/about/guidelines.xhtml

maps with clear details about access. Previous work has highlighted that this is often new and previously un-thought about information for participants [27]. The network maps, description of individual networks, preferences, and the local and online resources identified as relevant by individuals can be printed to keep or re-accessed online later via a personalised Genie page [40, 41]. Two weeks after the intervention all Genie users receive a phone call from the facilitator and alternative or additional engagement activities are discussed. The follow up call takes up to 10-15 minutes.

Wait-list control group

All participants allocated to the control group will be offered the opportunity to use the Genie intervention with a facilitator once the 6-month follow-up has been completed to avoid increasing inequalities as a result of the study, particularly for participants living in marginalized and deprived domestic situations.

Patient and Public Involvement

Several of our partner organisations were involved in the development of the study and protocol, particularly contributing to understanding methodological issues around identifying participants. We will continue to work closely with all stakeholders in a pragmatic and flexible way to assess implementation issues throughout the study. PPI representatives were consulted in the development phase of the study, as well as discussion with the CLAHRC Wessex Wiserd group, and prior Genie engagement work. In addition, further PPI representatives have been included in the trial management group, and we have consulted with the user-led McPin organisation, who are represented on our Steering committee. We will involve our PPI representatives in the interpretation of the findings from our studies, particularly those of user views.

Outcomes

For peer review only - http://bmjopen.bmj.com/site/about/guidelines.xhtml

The primary outcome of the trial will be the SF-12 Mental Health composite scale score at 6-month follow-up between the intervention and control group using a mixed effects model (accounting for clustering within facilitators and organisation).

Secondary outcomes will include:

- SF-12 Mental Health composite scale score between the intervention and control group at 3-month follow-up
- SF-12 Physical Health composite score between intervention and control groups at 6-month follow-up
- Loneliness between intervention and control groups at 3 and 6-month follow-up measured using the De Jong Loneliness scale [42] and the Campaign against loneliness measure [43]
- Social isolation between intervention and control groups at 3 and 6-month follow-up measured using the Duke Social Support index [44]
- Wellbeing measured using Warwick Edinburgh Mental Well-being scale (SWEMWBS) [45]
- Participant engagement with new activities

Economic evaluation measures will include:

- QALYs (incremental QALYs) between intervention and control at 6 months, with health related quality of life calculated using the SF-6D utility algorithm (derived from SF-12 data) [46]
- Incremental costs of public sector resource between intervention and control at 6 months
- Cost utility (expressed in terms of Cost/QALY and Cost/year of sufficient capability)
- Capability wellbeing measured using the ICEpop CAPability Measure for Adults (ICECAP-A) scores between intervention and control at 6 months [47]

For peer review only - http://bmjopen.bmj.com/site/about/guidelines.xhtml

For peer review only

Process evaluation measures will include:

- Participant perceived collective efficacy measured using the Collective Efficacy in Networks Scale (CENS) [48] and social support using the SPA [49]
- Perceptions of loneliness measured using a modified version of the Brief Illness Perception questionnaire (modified B-IPQ) [50]

Intervention group only:

- Social network composition change measured using Genie social network mapping (intervention group only at 3-months)

Study Endpoints:

At 3- and 6-months after enrolment in to the study, patients will be invited to complete follow-up assessments. They may do this independently or with the assistance of the facilitator or a research team member (which may include online, on paper or over the phone). All follow-up assessments will be recorded no earlier than two weeks before the follow-up date and no later than six weeks after the follow-up date**.**

Measures:

See Table2 for full details of study measures.

Table 2: Measures and schedule of observations within the PALS study

	**Time point (month)**		
**Measure**	Baseline	3 month follow-up	6 month follow-up
Socio-demographic measures	X		
**Patient self-report measures (both groups)**			
SF-12 Mental Health	X	X	X
SF-12 Physical Health	X	X	X
Loneliness (De Jong Scale)	X	X	X

For peer review only - http://bmjopen.bmj.com/site/about/guidelines.xhtml

For peer review only

Social isolation (Duke Social Support index)	X	X	X
Campaign to End Loneliness scale	X	X	X
Collective efficacy (CENS)	X	X	X
Social support (SPA)	X	X	X
Warwick Edinburgh Mental Well-being scale (SWEMWBS)	X	X	X
Perceptions of loneliness (modified B-IPQ)	X	X	X
Participant engagement with new activities	X	X	X
**Patient measures (network mapping, intervention group only)**			
Social network composition change	X	X	
**Economic measures**			
SF-6D	X	X	X
Capability wellbeing (ICECAP-A)	X	X	X
Health and social care use	X	X	X
**Process evaluation**			
Qualitative interviews with participants	X	X	X
Qualitative interviews with facilitators and stakeholders	X	X	X
Observations of facilitation	X		
Community staff observations of impact	X	X	X

Statistical analysis

All analyses will emphasise estimation and confidence intervals over hypothesis testing, and will be conducted as intention-to-treat. Missing data will be assumed to be missing at random, unless accounting for more than 10% of the sample; if missingness is above this rate, approaches for dealing with missing data (e.g. multiple imputation) will be discussed within the research team. Missingness will be reported for each arm and summaries of baseline characteristics of those lost to follow-up and those not will be used to judge potential sources of bias.

For peer review only - http://bmjopen.bmj.com/site/about/guidelines.xhtml

For peer review only

Baseline socio-demographic data will be summarised within randomised arms using appropriate descriptive measures; likewise, all outcome measures will be summarised by arm at each time-point. We will produce a forest plot of estimated effects for each outcome within each organisation to explore any variability in the impact of the intervention.

The primary analysis will involve a mixed effects model (pending the model meeting the associated assumptions) comparing groups on SF-12 at six months. The model will include a random intercept for facilitator and organisation, with participants clustered within facilitators clustered within organisation (hence a three-level model), and control for baseline SF-12. This analysis will be complemented by an analysis using the same framework but with SF-12 as the outcome and a random coefficient for time, where repeated measurements are clustered within participants (hence a four-level model).

Non-response bias (i.e., where a particular group of participants are unavailable or refuse to participate) will be reduced by taking steps to increase the initial response rate and reduce drop-out over the course of the study.

Economic analysis

The primary analysis will be a cost-utility analysis from a public sector perspective, with a primary outcome of cost/QALY at 6 months. Health related quality of life will be collected via SF-12 at baseline, 3 and 6 months, with utilities being derived by application of the SF-6D scoring algorithm [46]. In addition, scored values from the capability wellbeing measure (ICECAP-A) [47, 51] will enable a secondary cost-utility analysis [47]. The use of ICECAP–A is planned to explore non-health attributes (specifically capabilities) that might be important to this population, thus allowing for a broader measurement of wellbeing than might be captured by SF-6D. While the comparative data collected on both measures may

For peer review only - http://bmjopen.bmj.com/site/about/guidelines.xhtml

inform future studies in similar populations, it will also provide decision makers with richer information than would be obtained by a single generic HRQoL measure.

Intervention delivery resource use will be recorded on proformas designed to capture cost categories (e.g. trainer time, pay scale, intervention setting, facilitator travel costs). Additionally, at baseline, 3 and 6 months resource use will be collected directly from participants using a questionnaire designed to capture health care, social service and other public sector service use, as well as participant service use (i.e. participant and carer costs). An exploratory analysis will use a societal perspective providing decision makers with evidence to inform judgements on what, , in the broadest sense, is optimal for society [52]. The analysis of costs from a societal perspective will therefore provide detail on the cost-shifts within sectors (e.g. health compared to social care).. All analysis will follow practice guidelines [53-55], including those related to public health and/or complex interventions specifically [56-58]. Cost-utility analysis will also allow for the construction of cost-effectiveness acceptability curves to assess whether the intervention is cost-effective at a range of payer thresholds [59]. Sub-group analysis will be carried out in order to inform policy makers' decision making with respect to the targeting of the intervention. Such sub-group analyses (for instance looking at intervention effects in different groups) will be planned prospectively, and quantitative analysis - foreseeably including mixed effects modelling to account for the clustered nature of the data [60] - will be set out as part of the statistical and health economic analysis plan. The economic evaluation will also be informed by the process evaluation in terms of considering how the contexts of this complex intervention relate to resource use and cost areas [61]. Such an explanatory focus will be taken throughout the study, with a view to interpreting study results and assessing study generalisability.

Qualitative process evaluation and analysis

For peer review only - http://bmjopen.bmj.com/site/about/guidelines.xhtml

For peer review only

The qualitative process evaluation will combine complementary components to seek to provide an in-depth understanding of the factors that facilitate individual, environmental and organisational factors that inhibit or promote the engagement, workability, integration, sustainability and scalability of a social network intervention for addressing loneliness in open settings. The process evaluation will consider the pre-implementation contexts and processes, as well as observing use of the intervention in practice to understand the dynamics of implementation (including how the facilitation and other elements work) to consider implications for scale-up and sustainability for the participating organisations. Concepts from the Consolidated Framework for Implementation Research (CFIR) [62] will be used to guide the identification of factors promoting or inhibiting the routine incorporation and embeddedness of a facilitated social network intervention. The Non-adoption, Abandonment, and Challenges to the Scale-up, Spread, and Sustainability (NASSS) framework will inform the evaluation of implementation because it has been designed to help predict and evaluate the success of a technology-supported health program, addressing concerns such as implementation, scale-up and sustainability [63]. An ethnographic approach making use of observations, interviews and documentary analysis will be used to capture the pre-implementation processes in order to explore the workability and integration of Genie in different community organisations. Following this, interviews will take place to explore engagement, sustainability and scalability. Participants will be sampled purposively based on description of circumstances of loneliness and socio-demographic factors (age, gender, locality) we will explore the experiences and meaning of loneliness with reference to social and personal circumstances (e.g. living and working arrangements) and situational contexts of loneliness (such as migration, separation, unemployment). This will be combined with exploration of how individual circumstances shape engagement with different elements of the intervention, how change is enacted and embedded into people's everyday lives and how this

For peer review only - http://bmjopen.bmj.com/site/about/guidelines.xhtml

involves other members of a person's network. We will describe the engagement and activities undertaken following the intervention including how links with new networks and resources are identified and made (navigation); how these are integrated (negotiation); and how new connections improve capacity to enact healthy behaviours, improve wellbeing or reduce isolation (collective efficacy). We will explore how facilitators felt about delivering Genie and how this might be adopted by their organisations as part of their practice. We will draw out new improvements and benefits specific to individual circumstances and existing use of health care services. Further interviews post-intervention will be conducted until 'saturation' (i.e. no significant new insights emerge).

ETHICS AND DISSEMINATION

Ethical approval

Ethical approval for the PALS study has been obtained from the South Central – Berkshire B ethics committee (reference: 15/SC/0245). All substantial amendments must be approved by the University ethics committee and NHS ethics committee responsible for the trial, in additional to approval by HRA. Investigators are kept up to date with relevant changes via regular management group meetings.

Data monitoring

The Programme Steering Committee is responsible for ensuring programme adherence to the protocol, and adherence to the requirements of the Guidelines for Good Clinical Practice. The trial may be subject to inspection and audit by University of Southampton, under their remit as sponsor, the trial coordinating centre as the Sponsor's delegate and other regulatory bodies.

Dissemination

The findings from PALS will be disseminated widely through peer-reviewed publications, scientific conferences and workshops. In addition, we will aim to disseminate through

For peer review only - http://bmjopen.bmj.com/site/about/guidelines.xhtml

multiple community pathways in collaboration with our partners and stakeholders (including local councils, NHS trusts and other local and national organisations) through interactive methods, such as targeted workshops, podcasts or blogs. If successful, we aim to aim to produce a user guide for applying Genie to loneliness and isolation.

For peer review only

For peer review only - http://bmjopen.bmj.com/site/about/guidelines.xhtml

Author contributions:

RB developed the initial idea for the study and obtained funding in collaboration with AR, SE, DC, IV, MCP, LY, RK, CB and the PALS study team. All authors have contributed to the protocol development. RB, TCB, JE and AR have led the trial preparations and development of training materials. IV has lead the development and modification of Genie. SE and DE led the statistical analysis planning, and KB and RK have led the health economics planning. JE, CB and AR have led the qualitative process evaluation planning. RB wrote the initial draft, all subsequent drafts were contributed to by all authors who have approved the final version.

Funding Statement: This protocol paper summarises independent research funded by the National Institute for Health Research (NIHR) under its Public Health Research programme (Grant Reference Number 16/08/41). The views expressed are those of the author(s) and not necessarily those of the NHS, the NIHR or the Department of Health.

Competing interests: None.

For peer review only

References

1. Yang, K. and C. Victor, *Age and loneliness in 25 European nations.* Ageing and Society, 2011. **31**(8): p. 1368-1388.
2. Seeman, T.E., *Health promoting effects of friends and family on health outcomes in older adults.* Am J Health Promot, 2000. **14**(6): p. 362-70.
3. Perissinotto, C.M., I. Stijacic Cenzer, and K.E. Covinsky, *Loneliness in older persons: a predictor of functional decline and death.* Arch Intern Med, 2012. **172**(14): p. 1078-83.
4. Victor, C.R. and K. Yang, *The prevalence of loneliness among adults: A case study of the United Kingdom.* Journal of Psychology 2012. **146**: p. 85-104.
5. Lasgaard, M., K. Friis, and M. Shevlin, *"Where are all the lonely people?" A population-based study of high-risk groups across the life span.* Soc Psychiatry Psychiatr Epidemiol, 2016. **51**(10): p. 1373-1384.
6. Victor, C.R., V. Burholt, and W. Martin, *Loneliness and Ethnic Minority Elders in Great Britain: An Exploratory Study.* Journal of Cross-Cultural Gerontology, 2012. **27**(1): p. 65-78.
7. ONS, *Loneliness - What characteristics and circumstances are associated with feeling lonely?*, O.o.N. Statistics, Editor. 2018.
8. Valtorta, N.K., et al., *Loneliness and social isolation as risk factors for coronary heart disease and stroke: systematic review and meta-analysis of longitudinal observational studies.* Heart, 2016.
9. Niedzwiedz, C.L., et al., *The relationship between wealth and loneliness among older people across Europe: Is social participation protective?* Prev Med, 2016. **91**: p. 24-31.
10. Conditions, D.L.T., *Long Term Conditions Compendium of Information* D.o. Health, Editor. 2012.
11. Coyle, C.E. and E. Dugan, *Social isolation, loneliness and health among older adults.* J Aging Health, 2012. **24**(8): p. 1346-63.
12. Luo, Y., et al., *Loneliness, health, and mortality in old age: a national longitudinal study.* Soc Sci Med, 2012. **74**(6): p. 907-14.
13. Cacioppo, J.T., et al., *Loneliness as a Specific Risk Factor for Depressive Symptoms: Cross-Sectional and Longitudinal Analyses.* Psychology and Aging

2006 **21**(1): p. 140 –151.

14. Theeke, L.A. and J. Mallow, *Loneliness and Quality of Life in Chronically Ill Rural Older Adults: Findings from a pilot study.* The American journal of nursing, 2013. **113**(9): p. 28-38.
15. Musich, S., et al., *Homebound older adults: Prevalence, characteristics, health care utilization and quality of care.* Geriatric Nursing, 2015. **36**(6): p. 445-450.
16. Shankar, A., et al., *Loneliness, social isolation, and behavioral and biological health indicators in older adults.* Health Psychol, 2011. **30**(4): p. 377-85.
17. Holt-Lunstad, J., T.B. Smith, and J.B. Layton, *Social Relationships and Mortality Risk: A Meta-analytic Review.* PLOS Medicine, 2010. **7**(7): p. e1000316.
18. Steptoe, A., et al., *Social isolation, loneliness, and all-cause mortality in older men and women.* Proceedings of the National Academy of Sciences, 2013. **110**(15): p. 5797-5801.
19. Holt-Lunstad, J., et al., *Loneliness and Social Isolation as Risk Factors for Mortality: A Meta-Analytic Review.* Perspectives on Psychological Science, 2015. **10**(2): p. 227-237.
20. Cohen, G.D., et al., *The impact of professionally conducted cultural programs on the physical health, mental health, and social functioning of older adults.* Gerontologist, 2006. **46**(6): p. 726-34.
21. Taube, E., et al., *Loneliness and health care consumption among older people.* Scand J Caring Sci, 2015. **29**(3): p. 435-43.

For peer review only

22. Qualter, P., et al., *Investigating Hypervigilance for Social Threat of Lonely Children.* Journal of Abnormal Child Psychology, 2013. **41**(2): p. 325-338.
23. Reblin, M. and B.N. Uchino, *Social and emotional support and its implication for health.* Curr Opin Psychiatry, 2008. **21**(2): p. 201-5.
24. Feng, X. and T. Astell-Burt, *What types of social interactions reduce the risk of psychological distress? Fixed effects longitudinal analysis of a cohort of 30,271 middle-to-older aged Australians.* Journal of Affective Disorders, 2016. **204**: p. 99-102.
25. NICE, *Mental wellbeing and independence for older people (QS137)*, D.o. Health, Editor. 2016, National Institute for Health and Care Excellence
26. Blakeman, T., et al., *Effect of information and telephone-guided access to community support for people with chronic kidney disease: randomised controlled trial.* PLoS One, 2014. **9**.
27. Kennedy, A., et al., *Implementing a social network intervention designed to enhance and diversify support for people with long-term conditions. A qualitative study.* Implementation Science, 2016. **11**(1): p. 27.
28. Reeves, D., et al., *The contribution of social networks to the health and self-management of patients with long-term conditions: a longitudinal study.* PLoS One, 2014. **9**.
29. Council, H.C., *Social isolation and loneliness in Hampshire: A health needs assessment*. 2016.
30. Ware, J., Jr., M. Kosinski, and S.D. Keller, *A 12-Item Short-Form Health Survey: construction of scales and preliminary tests of reliability and validity.* Med Care, 1996. **34**(3): p. 220-33.
31. Dickens, A.P., et al., *An evaluation of the effectiveness of a community mentoring service for socially isolated older people: a controlled trial.* BMC Public Health, 2011. **11**(1): p. 218.
32. Parker, S.L., et al., *Assessment of the minimum clinically important difference in pain, disability, and quality of life after anterior cervical discectomy and fusion: clinical article.* J Neurosurg Spine, 2013. **18**(2): p. 154-60.
33. Elley, C.R., et al., *Intraclass correlation coefficients from three cluster randomised controlled trials in primary and residential health care.* Aust N Z J Public Health, 2005. **29**(5): p. 461-7.
34. Littenberg, B. and C.D. MacLean, *Intra-cluster correlation coefficients in adults with diabetes in primary care practices: the vermont diabetes information system field survey.* BMC Med Res Methodol, 2006. **6**.
35. Blickem, C., et al., *The clinical and cost-effectiveness of the BRinging information and guided help together (BRIGHT) intervention for the self-management support of people with stage 3 chronic kidney disease in primary care: study protocol for a randomized controlled trial.* Trials, 2013. **14**.
36. Rogers, A., et al., *Social networks, work and network-based resources for the management of long-term conditions: a framework and study protocol for developing self-care support.* Implementation Sci, 2011. **6**.
37. Vassilev, I., et al., *Social networks, social capital and chronic illness self-management: a realist review.* Chronic Illness, 2011. **7**.
38. Vassilev, I., et al., *Social networks, the 'work'and work force of chronic illness self-management: a survey analysis of personal communities.* PloS One, 2013. **8**.
39. Thoits, P., *Mechanisms linking social ties and support to physical and mental health.* J Health Hum Behav, 2011. **52**.
40. Blickem, C., et al., *Linking people with long-term health conditions to healthy community activities: development of PLANS (patient-Led assessment for network support).* Health Expect, 2013. **16**.
41. Blickem, C., et al., *Aligning everyday life priorities with people's self-management support networks: an exploration of the work and implementation of a needs-led telephone support system.* BMC Health Services Research, 2014. **14**(1): p. 262.
42. Gierveld, J.D.J. and T.V. Tilburg, *A 6-Item Scale for Overall, Emotional, and Social Loneliness: Confirmatory Tests on Survey Data.* Research on Aging, 2006. **28**(5): p. 582-598.
43. loneliness, C.a., *MEASURING YOUR IMPACT ON LONELINESS IN LATER LIFE*.

For peer review only - http://bmjopen.bmj.com/site/about/guidelines.xhtml

44. Koenig, H.G., et al., *Abbreviating the Duke Social Support Index for use in chronically ill elderly individuals.* Psychosomatics, 1993. **34**(1): p. 61-9.
45. NHS Health Scotland, U.o.W.a.U.o.E., *Short Warwick Edinburgh Mental Well-Being Scale (SWEMWBS).* 2008.
46. Brazier, J.E. and J. Roberts, *The estimation of a preference-based measure of health from the SF-12.* Med Care, 2004. **42**(9): p. 851-9.
47. Flynn, T.N., et al., *Scoring the Icecap-a capability instrument. Estimation of a UK general population tariff.* Health Econ, 2015. **24**(3): p. 258-69.
48. Band, R., et al., *Development of a measure of Collective Efficacy within personal networks: a complement to self-efficacy in self-management support? .* Patient Educ Couns, In submission
49. Cutrona, C.E. and D.W. Russell, *The provisions of social relationships and adaptation to stress.* Advances in personal relationships, 1987. **1**(1): p. 37-67.
50. Broadbent, E., et al., *The brief illness perception questionnaire.* J Psychosom Res, 2006. **60**(6): p. 631-7.
51. Al-Janabi, H., T. N Flynn, and J. Coast, *Development of a self-report measure of capability wellbeing for adults: the ICECAP-A.* Quality of Life Research, 2012. **21**(1): p. 167-176.
52. Jonsson, B., *Ten arguments for a societal perspective in the economic evaluation of medical innovations.* Eur J Health Econ, 2009. **10**(4): p. 357-9.
53. NICE, *Guide to the Methods of Technology appraisal*. 2013
54. Gold, M.R., *Cost-effectiveness in health and medicine*. Vol. xxiii. 1996, New York: Oxford University Press.
55. Glick, H., *Economic evaluation in clinical trials*. Vol. x,. 2007, New York: Oxford University Press.
56. Weatherly, H., et al., *Methods for assessing the cost-effectiveness of public health interventions.* key challenges and recommendations, 2009. **93**(2-3): p. 85-92.
57. Craig, P., et al., *Developing and evaluating complex interventions: The new Medical Research Council guidance.* International Journal of Nursing Studies, 2013. **50**(5): p. 587-592.
58. Payne, K. and A. Thompson, *Economic evaluations of complex interventions.* , in *Complex Interventions in Health: An Overview of Research Methods*, D.A. Richards and I.R. Hallberg, Editors. 2015, Routledge: Abingdon, UK. p. 248–257.
59. Fenwick, E., B.J. O'Brien, and A. Briggs, *Cost-effectiveness acceptability curves--facts, fallacies and frequently asked questions.* Health Econ, 2004. **13**(5): p. 405-15.
60. Sculpher, M.J., et al., *Generalisability in economic evaluation studies in healthcare: a review and case studies.* Health Technol Assess, 2004. **8**(49): p. iii-iv, 1-192.
61. Anderson, R. and R. Hardwick, *Realism and resources: Towards more explanatory economic evaluation.* Evaluation (Lond), 2016. **22**(3): p. 323-341.
62. Damschroder, L.J., et al., *Fostering implementation of health services research findings into practice: a consolidated framework for advancing implementation science.* Implementation Science, 2009. **4**(1): p. 50.
63. Greenhalgh, T., et al., *Beyond Adoption: A New Framework for Theorizing and Evaluating Nonadoption, Abandonment, and Challenges to the Scale-Up, Spread, and Sustainability of Health and Care Technologies.* J Med Internet Res, 2017. **19**(11): p. e367.

For peer review only - http://bmjopen.bmj.com/site/about/guidelines.xhtml

Figure 1: PALS study flow diagram

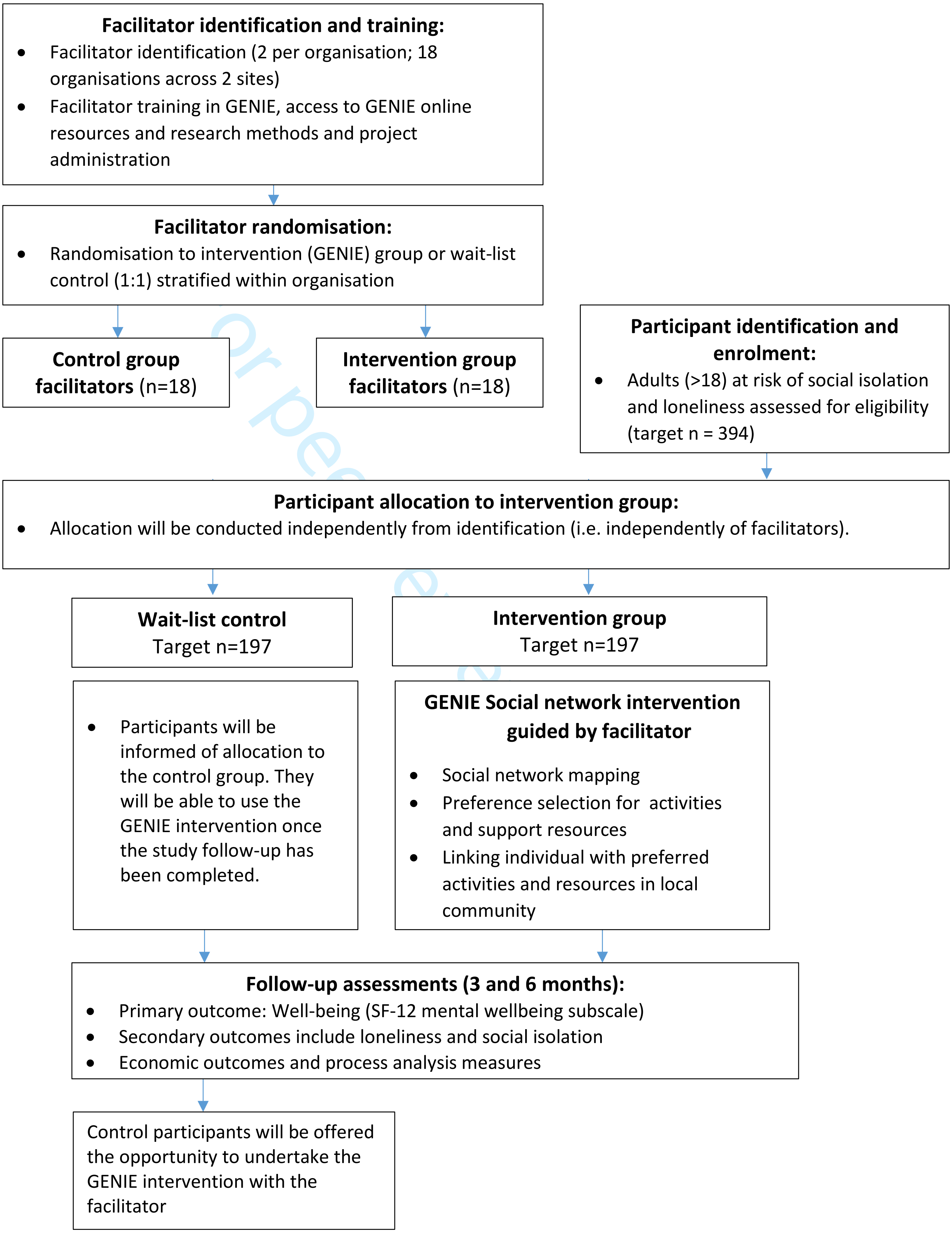

For peer review only - http://bmjopen.bmj.com/site/about/guidelines.xhtml

SPIRIT
STANDARD PROTOCOL ITEMS: RECOMMENDATIONS FOR INTERVENTIONAL TRIALS

SPIRIT 2013 Checklist: Recommended items to address in a clinical trial protocol and related documents*

Section/item	Item No	Description	Addressed on page number
**Administrative information**			
Title	1	Descriptive title identifying the study design, population, interventions, and, if applicable, trial acronym	1
Trial registration	2a	Trial identifier and registry name. If not yet registered, name of intended registry	3
	2b	All items from the World Health Organization Trial Registration Data Set	Not Applicable
Protocol version	3	Date and version identifier	1
Funding	4	Sources and types of financial, material, and other support	20
Roles and responsibilities	5a	Names, affiliations, and roles of protocol contributors	1
	5b	Name and contact information for the trial sponsor	No space to add
	5c	Role of study sponsor and funders, if any, in study design; collection, management, analysis, and interpretation of data; writing of the report; and the decision to submit the report for publication, including whether they will have ultimate authority over any of these activities	20
	5d	Composition, roles, and responsibilities of the coordinating centre, steering committee, endpoint adjudication committee, data management team, and other individuals or groups overseeing the trial, if applicable (see Item 21a for data monitoring committee)	No space to add

For peer review only

Introduction

Background and rationale	6a	Description of research question and justification for undertaking the trial, including summary of relevant studies (published and unpublished) examining benefits and harms for each intervention	5/6
	6b	Explanation for choice of comparators	12
Objectives	7	Specific objectives or hypotheses	6
Trial design	8	Description of trial design including type of trial (eg, parallel group, crossover, factorial, single group), allocation ratio, and framework (eg, superiority, equivalence, noninferiority, exploratory)	6/7

Methods: Participants, interventions, and outcomes

Study setting	9	Description of study settings (eg, community clinic, academic hospital) and list of countries where data will be collected. Reference to where list of study sites can be obtained	6/7
Eligibility criteria	10	Inclusion and exclusion criteria for participants. If applicable, eligibility criteria for study centres and individuals who will perform the interventions (eg, surgeons, psychotherapists)	7/8
Interventions	11a	Interventions for each group with sufficient detail to allow replication, including how and when they will be administered	10/11
	11b	Criteria for discontinuing or modifying allocated interventions for a given trial participant (eg, drug dose change in response to harms, participant request, or improving/worsening disease)	Not Applicable
	11c	Strategies to improve adherence to intervention protocols, and any procedures for monitoring adherence (eg, drug tablet return, laboratory tests)	Not Applicable
	11d	Relevant concomitant care and interventions that are permitted or prohibited during the trial	Not Applicable
Outcomes	12	Primary, secondary, and other outcomes, including the specific measurement variable (eg, systolic blood pressure), analysis metric (eg, change from baseline, final value, time to event), method of aggregation (eg, median, proportion), and time point for each outcome. Explanation of the clinical relevance of chosen efficacy and harm outcomes is strongly recommended	12-14
Participant timeline	13	Time schedule of enrolment, interventions (including any run-ins and washouts), assessments, and visits for participants. A schematic diagram is highly recommended (see Figure)	8/9

For peer review only - http://bmjopen.bmj.com/site/about/guidelines.xhtml

Sample size	14	Estimated number of participants needed to achieve study objectives and how it was determined, including clinical and statistical assumptions supporting any sample size calculations	9
Recruitment	15	Strategies for achieving adequate participant enrolment to reach target sample size	7
**Methods: Assignment of interventions (for controlled trials)**			
Allocation:			
Sequence generation	16a	Method of generating the allocation sequence (eg, computer-generated random numbers), and list of any factors for stratification. To reduce predictability of a random sequence, details of any planned restriction (eg, blocking) should be provided in a separate document that is unavailable to those who enrol participants or assign interventions	8
Allocation concealment mechanism	16b	Mechanism of implementing the allocation sequence (eg, central telephone; sequentially numbered, opaque, sealed envelopes), describing any steps to conceal the sequence until interventions are assigned	8
Implementation	16c	Who will generate the allocation sequence, who will enrol participants, and who will assign participants to interventions	8
Blinding (masking)	17a	Who will be blinded after assignment to interventions (eg, trial participants, care providers, outcome assessors, data analysts), and how	8
	17b	If blinded, circumstances under which unblinding is permissible, and procedure for revealing a participant's allocated intervention during the trial	Not applicable
**Methods: Data collection, management, and analysis**			
Data collection methods	18a	Plans for assessment and collection of outcome, baseline, and other trial data, including any related processes to promote data quality (eg, duplicate measurements, training of assessors) and a description of study instruments (eg, questionnaires, laboratory tests) along with their reliability and validity, if known. Reference to where data collection forms can be found, if not in the protocol	15
	18b	Plans to promote participant retention and complete follow-up, including list of any outcome data to be collected for participants who discontinue or deviate from intervention protocols	14

Data management	19	Plans for data entry, coding, security, and storage, including any related processes to promote data quality (eg, double data entry; range checks for data values). Reference to where details of data management procedures can be found, if not in the protocol	No space to add
Statistical methods	20a	Statistical methods for analysing primary and secondary outcomes. Reference to where other details of the statistical analysis plan can be found, if not in the protocol	15
	20b	Methods for any additional analyses (eg, subgroup and adjusted analyses)	No space to add
	20c	Definition of analysis population relating to protocol non-adherence (eg, as randomised analysis), and any statistical methods to handle missing data (eg, multiple imputation)	No space to add
**Methods: Monitoring**			
Data monitoring	21a	Composition of data monitoring committee (DMC); summary of its role and reporting structure; statement of whether it is independent from the sponsor and competing interests; and reference to where further details about its charter can be found, if not in the protocol. Alternatively, an explanation of why a DMC is not needed	Not applicable
	21b	Description of any interim analyses and stopping guidelines, including who will have access to these interim results and make the final decision to terminate the trial	No space to add
Harms	22	Plans for collecting, assessing, reporting, and managing solicited and spontaneously reported adverse events and other unintended effects of trial interventions or trial conduct	No space to add/ not applicable
Auditing	23	Frequency and procedures for auditing trial conduct, if any, and whether the process will be independent from investigators and the sponsor	Not applicable
**Ethics and dissemination**			
Research ethics approval	24	Plans for seeking research ethics committee/institutional review board (REC/IRB) approval	18
Protocol amendments	25	Plans for communicating important protocol modifications (eg, changes to eligibility criteria, outcomes, analyses) to relevant parties (eg, investigators, REC/IRBs, trial participants, trial registries, journals, regulators)	No space to add

Consent or assent	26a	Who will obtain informed consent or assent from potential trial participants or authorised surrogates, and how (see Item 32)	10
	26b	Additional consent provisions for collection and use of participant data and biological specimens in ancillary studies, if applicable	Not applicable
Confidentiality	27	How personal information about potential and enrolled participants will be collected, shared, and maintained in order to protect confidentiality before, during, and after the trial	No space to add
Declaration of interests	28	Financial and other competing interests for principal investigators for the overall trial and each study site	20
Access to data	29	Statement of who will have access to the final trial dataset, and disclosure of contractual agreements that limit such access for investigators	No space to add
Ancillary and post-trial care	30	Provisions, if any, for ancillary and post-trial care, and for compensation to those who suffer harm from trial participation	Not applicable
Dissemination policy	31a	Plans for investigators and sponsor to communicate trial results to participants, healthcare professionals, the public, and other relevant groups (eg, via publication, reporting in results databases, or other data sharing arrangements), including any publication restrictions	19
	31b	Authorship eligibility guidelines and any intended use of professional writers	Not applicable
	31c	Plans, if any, for granting public access to the full protocol, participant-level dataset, and statistical code	No space to add
**Appendices**			
Informed consent materials	32	Model consent form and other related documentation given to participants and authorised surrogates	No space to add
Biological specimens	33	Plans for collection, laboratory evaluation, and storage of biological specimens for genetic or molecular analysis in the current trial and for future use in ancillary studies, if applicable	Not applicable

*It is strongly recommended that this checklist be read in conjunction with the SPIRIT 2013 Explanation & Elaboration for important clarification on the items. Amendments to the protocol should be tracked and dated. The SPIRIT checklist is copyrighted by the SPIRIT Group under the Creative Commons "Attribution-NonCommercial-NoDerivs 3.0 Unported" license.

For peer review only - http://bmjopen.bmj.com/site/about/guidelines.xhtml
